# Surface-Controlled TiO_2_ Nanocrystals with Catalytically Active Single-Site Co Incorporation for the Oxygen Evolution Reaction

**DOI:** 10.1021/jacs.5c05795

**Published:** 2025-05-27

**Authors:** Chang Liu, Soonho Kwon, Perrin Godbold, Grayson Johnson, Sooyeon Hwang, Chengjun Sun, Hua Zhou, William A. Goddard, Sen Zhang

**Affiliations:** 1 Department of Chemistry, 2358 University of Virginia, Charlottesville, Virginia 22904, United States; 2 Materials and Process Simulation Center, 6469 California Institute of Technology, Pasadena, California 91125, United States; 3 Center for Functional Nanomaterials, 8099 Brookhaven National Laboratory, Upton, New York 11973, United States; 4 X-ray Science Division, Advanced Photon Source, 1291 Argonne National Laboratory, Lemont, Illinois 60439, United States

## Abstract

The design of advanced electrocatalysts is often hindered by uncertainties in identifying and controlling the active surfaces and catalytic centers within heterogeneous materials. Here we present the synthesis of single-site Co catalysts, substitutionally doped into surface-controlled TiO_2_ anatase nanocrystals, aimed at enhancing the oxygen evolution reaction (OER). Grand canonical quantum mechanics calculations reveal that the kinetics of the OER, following an adsorbate evolution mechanism, is markedly influenced by the coordination environment of Co. The simulations suggest significantly higher turnover frequencies when Co is doped into the (001) surface of TiO_2_ compared to the (101) surface. Consistent with the computational findings, experimental results show that Co-doped TiO_2_ (Co-TiO_2_) nanoplates with selectively exposed {001} surfaces exhibit enhanced current densities and turnover frequencies compared to Co-TiO_2_ nanobipyramids with {101} surfaces. This study highlights the synergy between theoretical calculations and precision synthesis in the development of more effective catalysts.

## Graphical abstract

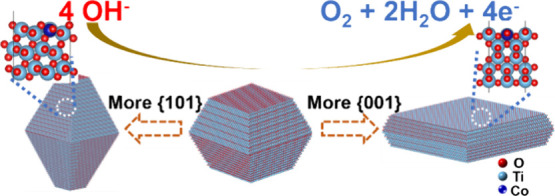

## Introduction

Creating an effective catalyst for the oxygen evolution reaction (OER) is essential for advancing various renewable technologies such as water electrolysis, metal air batteries,^1–4^ and regenerative fuel cells.^5,6^ Significant research efforts have been invested in both homogeneous and heterogeneous catalysis to elucidate the mechanisms and develop active materials for the OER. Homogeneous molecular catalysts are relatively easy to analyze regarding their active site structures, which facilitates a deeper understanding of the catalytic processes.^7^ However, these catalysts often face practical challenges, including limited durability, lower current densities, and issues with crossover in devices.^8–10^ On the other hand, heterogeneous nanomaterials have shown potential to surpass these obstacles, exhibiting enhanced catalytic performance.^11–16^ Yet, they typically lack the precisely defined active sites found in homogeneous catalysts.^17^ Even with uniform sizes and shapes, nanocrystals display a variety of active sites with differing levels of structural clarity.^18^ Single-atom catalysts appear promising in addressing these issues; still, many are supported by poorly defined materials, increasing the inhomogeneity and complicating the full characterization of their active sites.^19–21^

These challenges also extend to the computational simulation and prediction of optimal catalysts. While many heterogeneous electrochemical reactions in aqueous solutions have been described primarily by proton-coupled electron transfer,^22,23^ they also encompass chemical reactions, including water nucleophilic attacks^24–27^ and direct oxo coupling^28,29^ during the OER. Despite the relatively smaller potential dependence of these chemical reactions compared to redox reactions, they can lead to significant differences in rate constants under working conditions. Quantum Mechanics (QM) can provide the atomistic mechanisms underlying electrocatalysis, but the QM must be at constant potential for reactant, transition state, and product, whereas standard QM has only constant number of electrons. Thus, we need to apply Grand Canonical Quantum Mechanics (GCQM) to maintain a fixed electrochemical potential during the reaction steps.^30–32^ In this strategy, the grand potential is deduced indirectly by Legendre transforming the Helmholtz free energy, obtained from calculations with a series of fixed numbers of electrons (denoted Grand Canonical Potential Kinetics, GCP-K).^33^ This GCP-K has been used to accurately predicted the OER^34,35^ and CO_2_ reduction kinetics.^36,37^ A key requirement for correlating computational outcomes with experimental data is the detailed characterization of catalytic sites at the atomic level, a task that remains particularly challenging for heterogeneous catalytic materials.

This article explores the impact of coordination structure on the catalytic activity of single-site Co centers in the OER, achieved by manipulating surface facets of Co-doped TiO_2_ (Co-TiO_2_) anatase nanocrystals. Our prior work developed a technique for embedding single-site Co active centers into well-defined nanocrystal surfaces via substitutional doping, which yielded TiO_2_ brookite-phase (210) surfaces featuring atomically dispersed Co sites.^35^ This precise configuration of single-site Co facilitated a comprehensive integration of experimental and theoretical methods, with GCQM calculations of OER turnover frequencies (TOFs) closely mirroring experimental findings (13.7 s^–1^ via calculation and 6.6 s^–1^ in the experiment at 300 mV overpotential). This underscores the potential of further modulating Co single-site coordination structures to enhance catalyst properties for the OER. In the present study, we synthesized Co-TiO_2_ nanoplates and nanobipyramids to control the distribution of {001} and {101} surface facets. The nanoplates, characterized by a higher prevalence of {001} surfaces, exhibited improved performance compared to the nanobipyramids. Structural characterizations confirmed the single-site Co configurations, and our GCQM calculations aligned excellently with the experimental electrocatalytic results, confirming a significant enhancement in OER kinetics on the Co-TiO_2_ (001) surface relative to the (101) surface. Moreover, the calculations revealed that the Co-TiO_2_ (001) surface promotes the formation of five-coordinated Co sites under electrochemical conditions. This low-coordination environment is likely responsible for the enhanced catalytic activity observed, in contrast to the less active six-coordinated Co site on the Co-TiO_2_ (101) surface, suggesting the critical role of low-coordination sites in facilitating OER kinetics.

## Results and Discussion

### Synthesis and Characterization of Co-TiO_2_ Nanocrystals

The shape-controlled synthesis of Co-TiO_2_ anatase-phase nanocrystals was conducted using a colloidal method that involved the thermal decomposition of Co and Ti precursors in a solution containing 1-octadecene (ODE), oleic acid (OAc), and oleylamine (OAm). This process was a modification of previously reported methods.^35^ The hydrolysis of the Ti precursor occurred when small quantities of water released from OAm and OAc at elevated temperatures, leading to the formation of TiO_2_ nanocrystals. Earlier research documented that the adjustment of TiCl_4_ and TiF_4_ precursor ratios during synthesis, aided by halide ion modulators, controlled the anisotropic growth of TiO_2_, resulting in two distinct nanocrystal morphologies.^38^ Specifically, a TiCl_4_ to TiF_4_ ratio of 4:1 led to the creation of TiO_2_ nanobipyramids, while a reversed ratio of 1:4 resulted in TiO_2_ nanoplates (Figure 1A, S1). In our investigation, the use of mixed Co and Ti precursors for colloidal synthesis enabled the effective doping of Co into the anatase-phase TiO_2_ nanocrystals, producing Co-TiO_2_ nanoplates and nanobipyramids.

**Figure 1:**
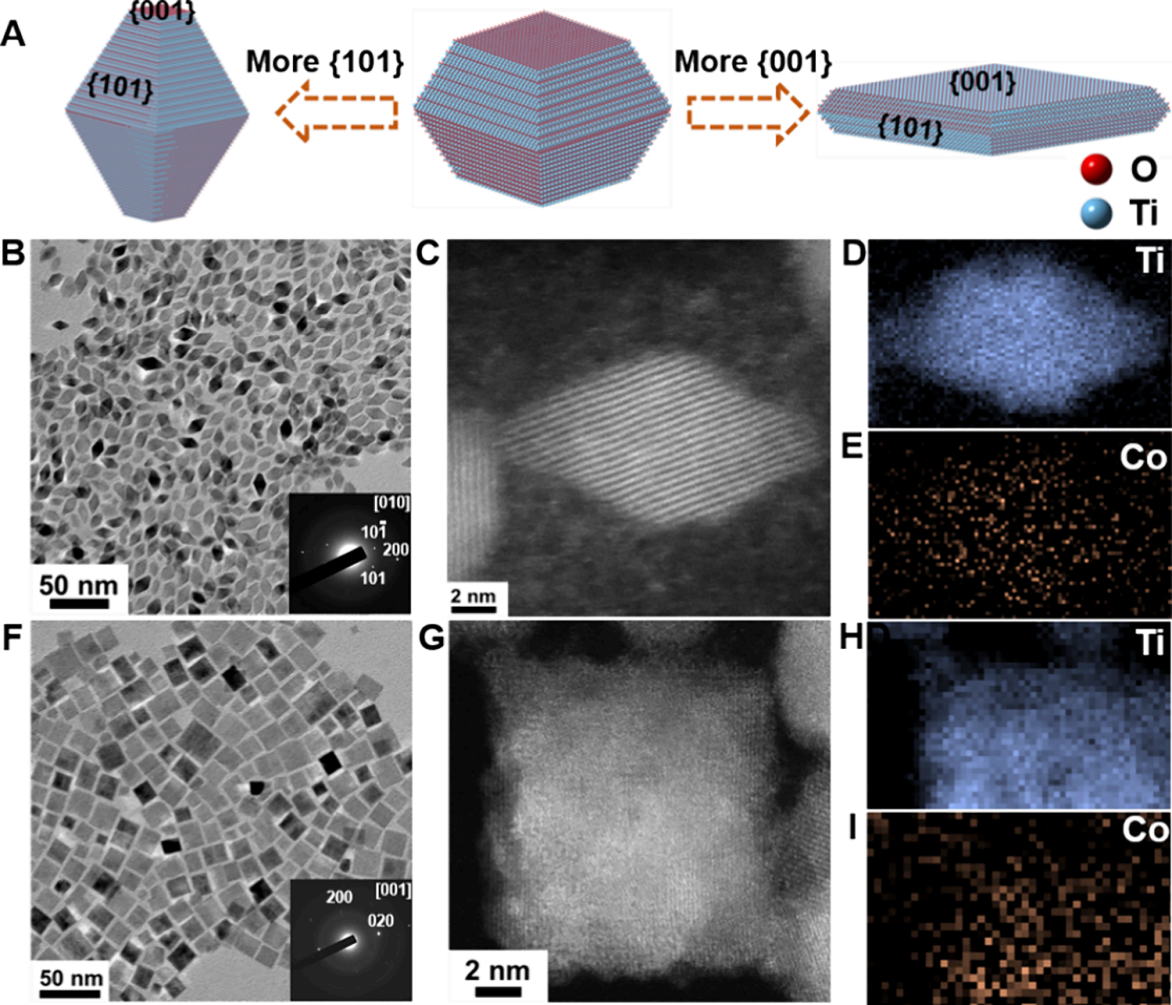
Morphology and structure characterizations of as-synthesized Co-TiO_2_ nanobipyramids and nanoplates. (A) Schematic illustrations displaying how varying the prevalence of {001} and {101} surface facets of Co-TiO_2_ nanocrystals results in nanobipyramid and nanoplate shapes; (B) TEM image of Co-TiO_2_ nanobipyramids (inset shows the SAED pattern of a single nanobipyramids); (C–E) representative HAADF-STEM image of a Co-TiO_2_ nanobipyramid and Ti and Co EELS elemental mapping; (F) TEM image of Co-TiO_2_ nanoplates (inset shows the SAED pattern of a single nanoplate); (G–I) representative HAADF-STEM image of a Co-TiO_2_ nanoplate and Ti and Co EELS elemental mapping.

Figure 1B, S2 shows the bipyramidal morphology of Co-TiO_2_, with a length of 17.0 ± 2.0 nm and a width of 9.3 ± 0.8 nm, and Figure 1F, S3 depicts the morphology of Co-TiO_2_ nanoplates, with dimensions of 5.8 ± 0.5 nm in length (thickness) and 16.7 ± 1.5 nm in width. High-angle annular dark-field scanning transmission electron microscopy (HAADF-STEM) analysis of Co-TiO_2_ nanobipyramids reveals a characteristic single crystal structure, displaying the {101} surfaces on the side planes and the {001} surfaces on the top and bottom (Figure 1C). Meanwhile, the nanoplates exhibit a predominant {001} surface complemented by the {101} side facets. The uniform distribution of Co dopants across both morphologies has been verified through electron energy loss spectroscopy (EELS) and elemental mapping (nanobipyramids in Figure 1 **D,** [Fig fig1] **E**; nanoplates in Figure 1 **H,** [Fig fig1] **I**). Raman spectroscopy (Figure S4) was employed to verify the variation in surface exposure ratios of the {001} and {101} facets. A higher exposure of the (001) facet is evidenced by a decrease in the E_g_ peak intensity and an increase in the A_1g_ peak intensity, corresponding to the symmetric stretching vibration of O–Ti–O and the antisymmetric bending vibration of O–Ti–O, respectively.^39^ No additional peaks were observed following the substitution of Ti sites with Co, suggesting the absence of phase segregation or formation of Co clusters or nanoparticles.

The crystal structures of TiO_2_ and Co-TiO_2_ were analyzed using X-ray diffraction (XRD). The XRD patterns, shown in Figure 2A and S5, confirm the anatase phase of TiO_2_ in the as-synthesized nanocrystals, consistent with the tetragonal structure indexed to JCPDS No. 02–0387. Notably, the incorporation of Co dopant does not introduce any new diffraction peaks, suggesting that the anatase phase of TiO_2_ is preserved following Co doping.

**Figure 2:**
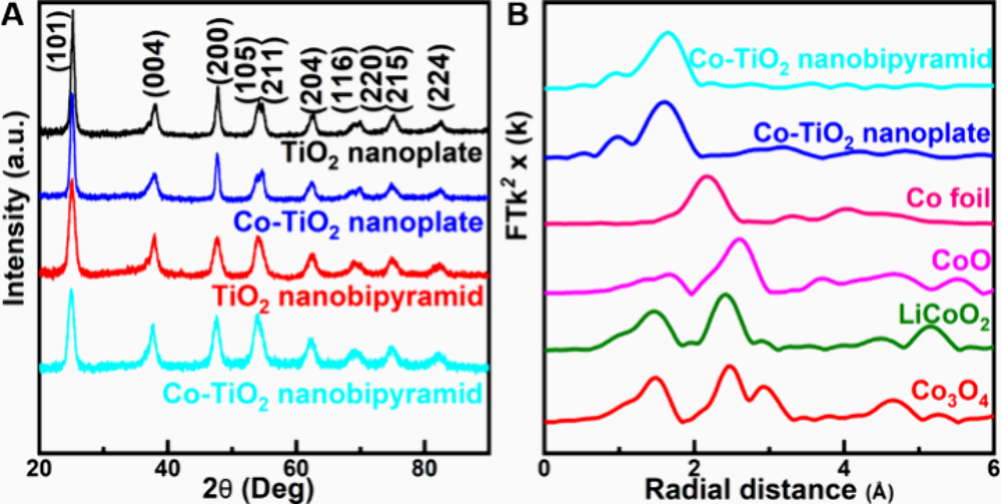
(A) XRD patterns of TiO_2_ and Co-TiO_2_ nanocrystals. (B) Co K-edge FT EXAFS spectra of Co-TiO_2_ nanobipyramids and nanoplates and other reference materials.

The levels of Co doping in both Co-TiO_2_ morphologies were adjusted by simply varying the amount of cobalt precursor used during synthesis, with the concentrations quantified using energy dispersive X-ray spectroscopy (EDS) (Figure S6). We found that the maximum Co doping level for anatase TiO_2_ nanocrystals was less than 4%, which is much lower than the approximately 10% observed for brookite TiO_2_ nanocrystals in our previous studies.^35,40^ To maintain consistency in our catalysis investigation, we controlled Co dopant concentration to be 3.9% for nanobipyramids and 3.5% for nanoplates. Transmission electron microscopy (TEM) and STEM images, presented in Figure 1 **and** Figure S7, confirm that the morphology of the Co-TiO_2_ nanocrystals closely resembles that of the undoped TiO_2_ nanocrystals.

The local coordination environment of the Co dopant was further investigated using Co K-edge extended X-ray absorption fine structure (EXAFS) spectroscopy. The EXAFS spectra for both the Co-TiO_2_ nanobipyramids and nanoplates are shown in Figure 2B. Notably, the spectra do not exhibit any scattering peaks at 2.17 Å, which would correspond to Co–Co interatomic bonds seen in the metallic cobalt foil reference. The predominant peak at 1.62 Å in the Co-TiO_2_ spectra is associated with the Co–O bond, suggesting that the first coordination shell characteristics of Co are similar for both morphologies (Figure S8, S9). Furthermore, the absence of Co–Co coordination peaks within the characteristic range of 2.1–2.4 Å, typical for cobalt oxide references such as CoO, LiCoO_2_, and Co_3_O_4_, confirms that there are no metallic cobalt clusters or secondary cobalt oxide phases present. This evidence supports the conclusion that cobalt dopants are atomically dispersed single sites, homogeneously integrated into the TiO_2_ matrix through substitution at Ti sites, further validating the uniformity of doping.

### Electrocatalytic Performance of Co-TiO_2_

The electrocatalytic performance of Co-TiO_2_ for the OER was evaluated using a three-electrode system in an O_2_-saturated 1 M KOH aqueous solution. The working electrode was fabricated by air-spraying a Co-TiO_2_ dispersion in hexane onto Toray carbon paper, followed by annealing at 200 °C for 12 h to remove organic surfactants. Linear sweep voltammetry (LSV), depicted in Figure 3A and S10, demonstrates that the Co-TiO_2_ nanocrystals exhibit significantly higher OER activity, in contrast to the negligible activity observed with pristine TiO_2_. This suggests that the catalytic activity of Co-TiO_2_ is attributable to the presence of Co in the nanocrystal surface. The overpotential required to achieve current densities of 10 mA cm^–2^_Geo_ and 100 mA cm^–2^_Geo_ (Geo indicates the current density was normalized over the electrode geometric area) for Co-TiO_2_ nanoplates was measured at 370 mV and 430 mV, respectively, while the value for Co-TiO_2_ nanobipyramids was 390 mV to reach a current density of 10 mA cm^–2^. Electron microscopy analysis indicates a variation in the abundance of the (001) and (101) facets between the two morphologies, which is likely the cause of their differing electrocatalytic performances.

**Figure 3:**
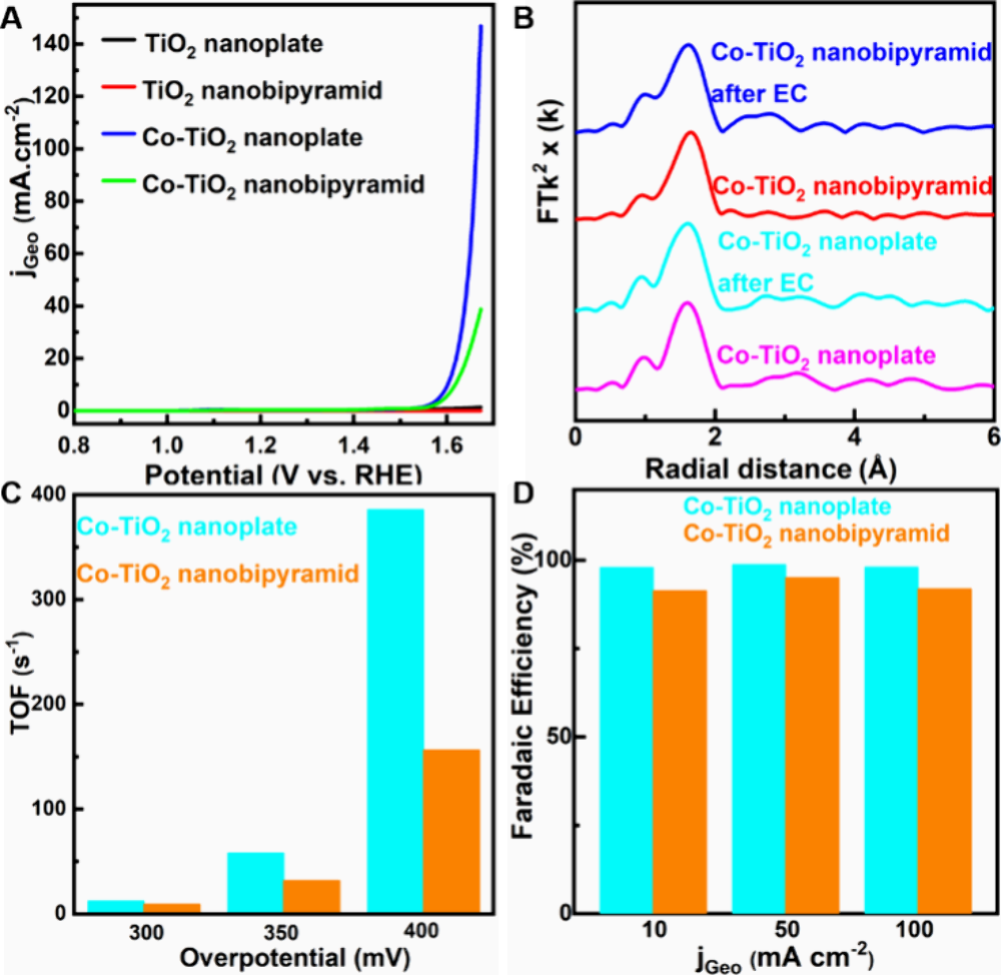
OER catalytic performance of Co-TiO_2_ nanocrystals in 1 M KOH aqueous electrolyte. (A) LSV plot for different catalysts. (B) Co K-edge EXAFS of Co-TiO_2_ nanocrystals before and after electrochemical tests. (C) TOFs of Co-TiO_2_ nanocrystals at different overpotentials. (D) FEs of Co-TiO_2_ nanocrystals at different current densities.

Ex-situ EXAFS analysis of the Co-TiO_2_ nanocrystals, as shown in Figure 3B, S11, and S12, confirmed that the chemical environment of the single-site Co remained stable after the OER tests. Furthermore, the Co EXAFS profiles aligned with those of Ti across all tests (Figure S13–S19, Table S1). Both in TiO_2_ and Co-TiO_2_, there was consistent agreement in the bond distances between Co–O and Ti–O within the first coordination shell and a similar scattering pattern between Co–Ti and Ti–Ti in the second coordination shell. These findings support the effective preservation of single-site Co within the anatase TiO_2_ lattice under OER conditions.

With this stable and reliable structure, it is possible to calculate the turnover frequency (TOF) using reasonable estimations. First, the ratio of (101) to (001) facets on each nanocrystal morphology was determined based on their geometric sizes as observed in TEM images. (Table S2, Figure S20). Then, the electrochemical active surface area (ECSA) of both the Co-TiO_2_ nanoplate and nanobipyramid electrodes was calculated from measurements of the electrochemical double-layer capacitance (Figure S21, S22). Subsequently, considering the exposure of Co within each TiO_2_ lattice, we estimated the quantity of single-site Co active centers from the ECSA. The TOF was calculated by normalizing the catalytic current density by the number of Co atoms present in each electrode, with results presented in Figure 3C. The Co-TiO_2_ nanoplates exhibited TOFs of 12.1 s^–1^ at 300 mV, 58.0 s ^–1^ at 350 mV, and 385.6 s^–1^ at 400 mV overpotential, whereas the Co-TiO_2_ nanobipyramid demonstrated lower TOFs of 9.3 s^–1^ at 300 mV, 31.6 s^–1^ at 350 mV, and 156.2 s^–1^ at 400 mV overpotential (Table S3). The activity reflects contributions from all active sites, and given the differing facet abundances in the two nanocrystals (Table S2 *),* the higher activity of Co-TiO_2_ nanoplates suggests that the (001) facet enables more favorable OER kinetics.

Faradaic efficiency (FE) analyses of the Co-TiO_2_ carbon paper electrodes were conducted using online gas chromatography (GC) to evaluate the OER efficiency. As depicted in Figure 3D, the FEs for O_2_ formation using the Co-TiO_2_ nanoplate catalyst exceeded 98% at current densities of 10, 50, and 100 mA cm^–2^. Comparable results were achieved with the Co-TiO_2_ nanobipyramid catalyst, which displayed a slightly lower FE of 92% at 100 mA cm^–2^. Chronoamperometric (CA) tests (Figure S23) confirmed the robust stability of the Co-TiO_2_ nanoplate catalyst, although there was a minor decay observed at higher current densities.

The TEM images and EXAFS spectra of the Co-TiO_2_ nanocrystals, which showed no significant morphological changes or spectral variations before and after OER testing (Figure S8–13, S24, S25), indicate that the structure of the catalysts remained intact. Given the well-defined and stable nature of these sites, we investigated further using representative models and GCQM calculations to elucidate the underlying mechanism for this marked difference in activity.

### GCQM Calculations

The anatase TiO_2_ crystal features chains of distorted TiO_6_ octahedra, resulting in a tetragonal structure (D_4h_,^19^ *I*4_1_/*amd*). The (001) terrace of TiO_2_ is characterized by unsaturated surface atoms that are five-coordinated titanium (Ti_5c_) with 2-fold bridge surface oxygen atoms (O_2c_). In contrast, the (101) surface exhibits six-coordinated titanium (Ti_6c_) along with two types of three-coordinate oxygen (O_3c_), as well as under-coordinated Ti_5c_ and O_2c_. Cobalt substitution preferentially occurs at the Ti_5c_ sites on the topmost layer rather than at the Ti_6c_ sites on the second layer, with an energy difference of 0.54 eV for the (001) facet and 0.13 eV for the (101) facet of TiO_2_ (Figure S26). Moreover, the Co substitution energy on the TiO_2_ (001) surface is only 0.19 eV lower than on the TiO_2_ (101) stoichiometric surface (Figure S27). Considering the small energy difference and our high-temperature synthesis condition, it is unlikely that Co doping is confined exclusively to the (001) facet within nanocrystals.

We constructed surface energy diagrams for undoped anatase TiO_2_ surfaces. Starting with TiO_2_ (001) and (101) surfaces covered by 1 ML of adsorbed water (H_2_O* 1 ML), the oxidation of H_2_O* progresses to *OH and subsequently to *O, where the surface energy depends on the applied potential, as illustrated in Figure S28. The Gibbs free energy for each state is summarized in Table S4. On the (001) surface, the oxidation of H_2_O* to *OH becomes thermodynamically favorable at potentials (U) higher than 1.92 V vs RHE, which is significantly above the operational potential for the OER. On the (101) surface, H_2_O* can directly oxidize to *O only at U > 2.45 V vs RHE. This finding confirms the negligible OER activity from the undoped TiO_2_.

It is known that anatase TiO_2_ demonstrates a preference for dissociative water adsorption on TiO_2_ (001) surface, while the Ti_5c_ site on the TiO_2_ (101) surface typically favors molecular adsorption.^41–43^ Indeed, we found that a monolayer of explicit water solvent on the Co-TiO_2_ (001) facet spontaneously reacts with the bridging oxo (O_b_), resulting in the formation of four hydroxyl groups at both Co and Ti sites, as detailed in the Figure S29. Conversely, only molecular water adsorption is observed on the (101) facet, as depicted in Figure 4C (State 1). This distinct difference in adsorption behavior between the (001) and (101) facets lead to notably different arrangements of surface and bond species at the active sites, even before applying any potentials.

**Figure 4:**
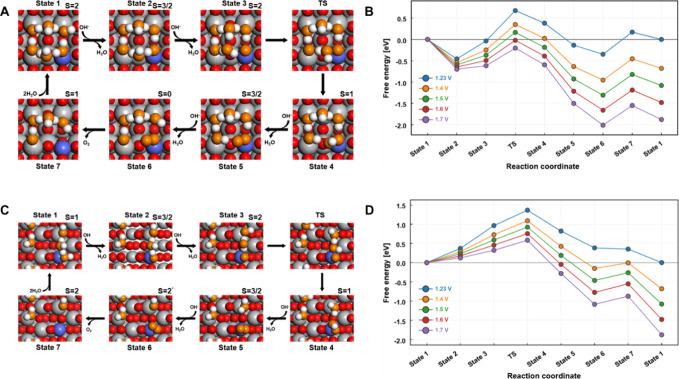
OER mechanism on Co-TiO_2_ surfaces. (A) Schematic illustration of OER mechanism on Co-TiO_2_ (001) surface. (B) Free-energy landscape for OER pathway (AEM) on Co-TiO_2_ (001) surface as a function of applied potential at pH = 14. (C) Schematic illustration of OER mechanism on the Co-TiO_2_ (101) surface. (D) Free-energy landscape for OER pathway (AEM) on Co-TiO_2_(101) surface shown as a function of applied potential at pH = 14. Each color in (A) and (C) represents a different element: blue (Co), gray (Ti), white (H), red (lattice O), orange (O of the surface water). The spin state of each system is indicated in the top-right corner of each configuration.

Moreover, our calculations reveal that during the dissociative water adsorption process, the Co-TiO_2_ (001) surface evolves to a five-coordinated Co configuration (including adsorbed intermediates such as *OH and *O) through the loss of a bridging oxygen atom between Co and an adjacent Ti atom (Figure S30). This coordination environment remains stable throughout all the states along the reaction pathway (Figure 4). In contrast, the Co-TiO_2_ (101) surface maintains a six-coordinated Co site (including adsorbed intermediates). This difference may arise from the lower surface energy of the TiO_2_ (101) surface, in contrast to the (001) surface, which exhibits a lower oxygen vacancy formation energy. Indeed, the (101) surface is the most thermodynamically stable facet of anatase TiO_2_.^44,45^

We conducted GCQM calculations on the (001) and (101) surfaces of Co-TiO_2_ to predict the TOFs and current densities as a function of applied potential. Based on our previous research on the brookite Co-TiO_2_, we hypothesized that the adsorbate evolution mechanism (AEM) was predominant in driving the OER activity.^35,46,47^ The free-energy landscapes depicting the seven elementary steps along OER process on Co-TiO_2_ at pH 14 as a function of potential are presented in Figure 4. The reaction progresses through four electrochemical oxidation steps, leading to a net reaction under our working condition: 4OH^–^ **→** 2H_2_O **+** 4e^–^ **+** O_2_.

Figure 4 **A and** [Fig fig4] **B** show the OER mechanism on the Co-TiO_2_ (001) surface. At 1.6 V vs RHE, the initial oxidation step involves the deprotonation of surface water at the Co (State 1), transforming it into Co-*OH (State 2), a transition that is energetically favorable by 0.62 eV. The specific capacitance of State 2 is determined to be 12.1 μF cm^–2^ within a potential range of 0.69 to 0.72 V, as calculated using a five-layer slab model with inversion symmetry (Figure S31). Subsequent deprotonation of the Co-*OH (State 2) results in the formation of a triplet Co^5+^ state bonded to an active terminal oxo species (State 3), with a reaction free energy (ΔG) of +0.15 eV. In this state, the four d-electrons are spread across three t_2g_-like orbitals, creating a triplet state characterized by electron delocalization between the singly occupied dp on Co and the doubly occupied pp on the oxo. This configuration imparts a radical character to the oxygen atom, having 0.77 e- according to Bader analysis, as shown in Figure S32.

In the third step of the process, the Co = O oxo bond in State 3 reacts with an adjacent H_2_O molecule, leading to the formation of a new O–O bond and the creation of Co-OOH in State 4.

Concurrently, an H atom from the H_2_O molecule is transferred to a neighboring Ti = O. The overall energy barrier for this reaction includes the thermodynamic barrier required to form the active oxygen species in State 3 and the kinetic barrier for the O–O coupling transition state (TS), resulting in a ΔG^‡^=0.63 eV. The subsequent two oxidation steps involve exothermic deprotonations. The transition from State 4 to State 5 results in an energy release of 0.82 eV, and the progression from State 5 to State 6 releases 0.43 eV. Subsequently, the transformation from State 6 to State 7 involves the endothermic release of O_2_, requiring an input of 0.47 eV. The cycle completes with a return from State 7 to State 1 by incorporating two water molecules, an exothermic reaction that releases 0.29 eV.

The mechanism of OER on the Co-TiO_2_ (101) surface is illustrated in Figure 4 **C and** [Fig fig4] **D**. Analogous to the (001) surface, the initial oxidation step, transitioning from State 1 to State 2, involves the deprotonation of surface-bound water, leading to the formation of Co–OH*. Different to this step on (001) surface, it requires an energy input of 0.18 eV at 1.6 V. The next deprotonation from State 2 to State 3, an energy uphill process by 0.28 eV, transforms Co–OH into the oxygenated Co = O species. This species also exhibits a radical character, as indicated by Bader analysis, showing an electron configuration of 0.83 e^–^, which is 0.06 e^–^ higher than that on the (001) surface (Figure S32). The step from State 3 to State 4 involves O–O coupling with an adjacent H_2_O molecule, leading to a free energy barrier of 0.30 eV at the TS, which is 0.11 eV lower than that on the (001) surface. Nevertheless, the overall barrier from the resting state at 0 to the TS is 0.76 eV, which is 0.13 eV higher than the 0.63 eV barrier predicted for the (001) surface.

The subsequent oxidation steps involve exothermic deprotonations, with an energy release of 0.80 eV as it transitions from State 4 to 5, and 0.73 eV from State 5 to 6. Following this, the progression from State 6 to 7 enables the release of O_2_ which is endothermic, consuming 0.22 eV. The final transition from State 7 back to State 1 includes the addition of two water molecules, which is an exothermic reaction releasing 0.93 eV of energy.

Based on the detailed energy landscapes of two Co-TiO_2_ surfaces, we calculate the TOFs for each of them employing the Eyring equation.

$$\mathrm{TOF}=\frac{k_{B}T}{h}\times \exp\left(\frac{\Delta G^{‡}}{k_{B}T}\right)$$

where *k*_*B*_ is the Boltzmann constant, T is temperature (298 K), and *h* is Planck’s constant. The free energy barrier, ΔG^‡^, is the free-energy difference between the TS of the O–O coupling step and the resting state (State 2 for (001) and State 1 for (101)). The current density is calculated based on the TOF assuming 25 at. % surface Co concentration for (001) and 12.5 at. % surface Co concentration for (101) surfaces according to our model system (Figure 4).

Figure 5A displays the comparison of the theoretical and experimental LSV plots, which have been normalized based on the ECSA. The predicted overpotential at 10 mA cm^–2^ on the (001) surface is 0.36 V, closely matching the experimental value of 0.40 V observed on the Co-TiO_2_ nanoplate. For the Co-TiO_2_ (101) surface, the theoretical overpotential for the oxygen evolution reaction (OER) at 10 mA cm^–2^ is 0.45 V, aligning with the experimental overpotential of 0.44 V on the bipyramid catalysts.

**Figure 5:**
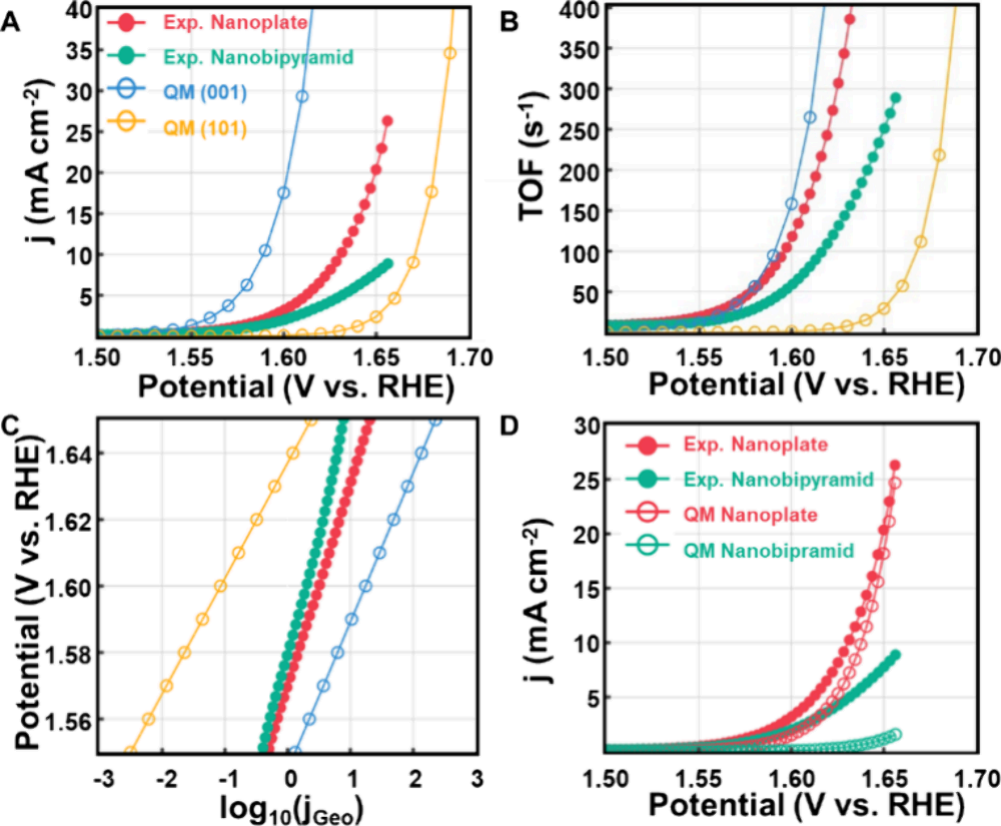
Comparison of the experimental LSV and GCQM predictions of OER on Co-TiO_2_. (A) Current density assuming 25% surface Co for (001) and 12.5% for (101). (B) Turnover frequency per Co site. (C) Tafel slope for experiments on the nanoplate and nanobipyramid catalysts compared with predicted for (001) surface and (101) surface. (D) Predicted current density assuming that the nanoplate is 51.6% (001) occupancy and that the nanobipyramid is 2.9% (001).

The theoretical TOFs at 1.6 V are estimated to be 295 s^–1^ for the (001) surface and 0.5 s^–1^ for the (101) surface, as summarized in Figure 5B. This demonstrates that the OER activity of the (001) facet is significantly higher, which is consistent with the elevated experimental TOFs observed in the nanoplates dominated by the (001) facet. Similarly, the theoretical Tafel slopes are determined to be 45 mV dec^–1^ for the (001) surface and 35 mV dec^–1^ for the (101) surface. These values can be compared with experiment on the nanoplate catalysts (62 mV dec^–1^) and nanobipyramid catalyst (76 mV dec^–1^) over the range from 1.55 to 1.67 V (Figure 5C).

The enhanced OER activity on the Co-TiO_2_ (001) surface compared to the (101) surface is likely attributed by the lower coordination environment of Co. As mentioned above, the five coordinated Co sites on (001) surface are stable across the OER pathway in our calculations. At the highest oxidation state (State 3), Co on the (001) surface can sustain a relatively lower oxidation state compared to that on the (101) surface due to the reduced oxygen coordination. This results in the smaller reaction barrier on the (001) surface relative to its corresponding resting state (State 2), requiring a lower overpotential than on the (101) surface, where the resting state is State1. It suggests that harnessing low-coordination Co sites holds great promise for enhancing OER activity.

Using the facet ratios of (001) and (101) surfaces from Table S2 and the Co-doping levels for both Co-TiO_2_ nanocrystals, the theoretical current density (*j*_*QM*_) for each catalyst can be calculated using the formula:

$$j_{QM}=j_{\left(001\right)}^{QM}\times f_{\left(001\right)}^{\exp}\times \frac{c_{Co}^{\exp}}{{c_{Co}}_{\left(001\right)}^{QM}}+j_{\left(101\right)}^{QM}\times f_{\left(101\right)}^{\exp}\times \frac{c_{Co}^{\exp}}{{c_{Co}}_{\left(101\right)}^{QM}}$$

Here *j*_(001)_^*QM*^ and *j*_(101)_^*QM*^ are the theoretical current densities for the respective surfaces, *f*_(001)_^*exp*^ and *f*_(101)_^*exp*^ denote the experimental fraction of facets, and *c*_*Co*__(001)_^*QM*^ and *c*_*Co*__(101)_^*QM*^ are surface Co concentrations for the slab model of the (001) and (101) surfaces, respectively. *c*_*Co*_^*exp*^ is the doping concentration for each catalyst. The surface Co concentrations were taken from experiment to be 3.5% for nanoplate and 3.9% for nanobipyramids catalysts. The experimental current density is normalized over the ECSA. This calculation facilitates a direct comparison between theoretical and experimental results for complete nanocrystals. Notably, the edge and corner sites were not considered in this model.

Figure 5D shows that the predicted current density for the nanoplate aligns exceedingly well with experimental data. However, the current density predicted for the nanobipyramid catalyst is lower than what is observed experimentally. By fitting the theoretical currents to experimental LSV through a linear combination of two *j*_*QM*_ from each facet, we found that the nanobipyramid containing 12% of (001) facet more accurately matches the experimental current density. Therefore, the discrepancy in the current density of the nanobipyramid likely stems from an underestimation of about 9% in the (001) facet’s contribution or from contributions of unaccounted edge sites and defects. For both catalysts, the contributions from the (101) facets are predicted to be too small at potentials less than 1.65 V (Figure 5 **A,** [Fig fig5] **B**).

Owing to the strongly correlated nature of cobalt oxidized systems, the calculated energetics in this work may be sensitive to the choice of exchange-correlation functional especially for the redox reaction at the active sites.^48^ A thorough investigation of this dependence is beyond the scope of the present study and should be addressed in future work.

## Conclusions

In summary, we developed anatase-phase Co-TiO_2_ nanoplates and nanobipyramids, each with varying ratios of (001) and (101) surface facets, and integrated uniformly dispersed single-site Co into TiO_2_. The resulting Co-TiO_2_ nanocrystals were assessed for OER performance in an alkaline electrolyte and demonstrated facet-dependent catalytic properties. Leveraging the well-defined atomic structures of the nanocrystals, we successfully conducted GCQM calculations to elucidate the nine-step OER reaction mechanisms in relation to the applied potentials for each catalyst. This modeling resulted in OER kinetics that closely matched experimental observations. Our findings indicate that the OER activity of the nanoplate and nanobipyramid catalysts is predominantly influenced by the (001) surfaces of the Co-TiO_2_, which achieve smaller overpotentials compared to the (101) surface. This study underscores the significant impact of single-site coordination environments on catalytic activity and the potential of low-coordination Co sites for enhancing OER kinetics. It also demonstrates the effectiveness of combining theoretical and experimental approaches to advance the mechanistic understanding of electrocatalysis.

## Supplementary Material





## Notes

Experimental section, Figures S1–S34, and Tables S1–S4 (PDF)
Geometry files with header information with energy, electrochemical potential, and total number of electrons (ZIP)

## †

Chemical Sciences Division, Oak Ridge National Laboratory, Oak Ridge, Tennessee 37831, United States
