# Supplementary material for: Surface-Controlled TiO2 Nanocrystals with Catalytically Active Single-Site Co Incorporation for the Oxygen Evolution Reaction
Source: J Am Chem Soc. 2025 May 27;147(22):19391–9. doi: 10.1021/jacs.5c05795 (PMC12147152; doi:10.1021/jacs.5c05795)
Supplement: Supplementary file 1 [file ja5c05795_si_001.pdf]

Supporting Information

Surface-Controlled TiO_2 Nanocrystals with Catalytically Active Single-Site Co Incorporation for the Oxygen Evolution Reaction

Chang Liu,[1,#] Soonho Kwon,[2,#] Perrin Godbold,[1] Grayson Johnson[1], Sooyeon Hwang,[3] Chengjun Sun,[4] Hua Zhou,[4] William A. Goddard III,[2,*] Sen Zhang[1,*]

[1] Department of Chemistry, University of Virginia, Charlottesville, Virginia 22904, USA;

[2] Materials and Process Simulation Center, California Institute of Technology, Pasadena, California 91125, USA;

[3] Center for Functional Nanomaterials, Brookhaven National Laboratory, Upton, New York 11973, USA;

[4] X-ray Science Division, Advanced Photon Source, Argonne National Laboratory, Lemont, Illinois 60439, USA.

[#] These authors contributed equally.

*Corresponding Authors:

Email for W.A.G.: wag@caltech.edu; ORCID:0000-0003-0097-5716

Email for S.Z.: sz3t@virginia.edu; ORCID: 0000-0002-1716-3741

Experimental

Chemicals and materials Titanium fluoride (TiF_4, 99%) was purchased from Alfa Aesar. Titanium chloride ($TiCl_4$, 99%) was procured from Fluka Analytical. Cobalt (II) acetylacetonate ($Co(acac)_2$, 99%) and 1-octadecene (ODE, 90%) were sourced from Acros Organics. Oleic acid (OAc, 90%) and oleylamine (OAm, 70%) were purchased from Sigma-Aldrich. Additional solvents, hexane (ACS Certified) and 2-propanol (IPA, ACS Certified), and KOH (ACS Certified) were purchased from Fisher Scientific. Toray carbon paper 060 was obtained from Fuel cell store.

Synthesis of Co-TiO_2 and TiO_2 nanoplates TiO_2 and Co-TiO_2 nanoplates were synthesized by typical colloidal synthesis with some modifications from our previous methods, conducted in moisture free condition using Schlenk line techniques. All solvents used in the synthesis underwent pre-drying at 90 °C under vacuum for 1 hour and were stored in a N_2-filled glovebox. For the synthesis of TiO_2 nanoplates, two titanium stock solutions, TiF_4 and $TiCl_4$, each containing 0.2 M of Ti^{4+} and 1 M of OAc in ODE, were prepared in the glovebox and stored for subsequent use. The titanium precursor was then prepared by mixing the $TiCl_4$ and TiF_4 stock solution in a volumetric ratio of 4/1 $TiF_4/TiCl_4$. A degassed system, containing 10 ml of ODE, 10 ml of OAm and 0.48 ml of OAc, was heated to 90 °C under vacuum for 1 hour and then cooled down to 60 °C in a N_2 atmosphere. 1.5 ml of Ti precursor solution was injected and the system was then heated to 290 °C at a ramp rate of 10 °C min^{-1}. After maintaining the reaction at 290 °C for 10 minutes, 8 ml of titanium precursor was slowly pumped into the reactor at a rate of 0.3 ml min^{-1}. Following removal of heating mantle and cooling to room temperature, the TiO_2 nanoplates were washed with IPA and separated via centrifugation at 8000 rpm for 8 minutes. This washing procedure was repeated twice to remove excess surfactants. The synthesis of Co-TiO_2 nanoplates followed a similar method. A Co-oleate ODE solution containing 0.2 M of $Co(acac)_2$ and 1.0 M of OAc was mixed

with the Ti-precursor solution at a Ti/Co ratio of 8/3 to form the precursor mixture. Subsequently, 1.5 ml of the precursor mixture was injected to pre-dried solvents (10 ml of ODE, 10 ml of OAm and 10 ml of OAc) at 60 ℃ in N_2. The reaction system was rapidly heated to 290 ℃ and held at the temperature for 10 minutes. 8 ml of the precursor mixture was slowly injected into the system at a rate of 0.3 ml min^{-1}. The Co-TiO_2 nanoplates were then purified and collected with the same method as for the TiO_2 nanoplates.

Synthesis of Co-TiO_2 and TiO_2 nanobipyramids TiO_2 nanobipyramids and Co-TiO_2 nanobipyramids were synthesized following a similar procedure to that of TiO_2 nanoplates and Co-TiO_2 nanoplates synthesis, albeit with a different ratio of TiF_4/$TiCl_4$ 1/4. The titanium precursor was prepared by mixing a stock solution of TiF_4 and $TiCl_4$ in a volumetric ratio of TiF_4/$TiCl_4$ 1/4. For the synthesis of TiO_2 nanobipyramids, 1.5 ml titanium precursor was injected into degassed solvents containing 10 ml of ODE, 10 ml of OAm and 0.48 ml of OAc at 60 ℃ under a N_2 atmosphere. Subsequently, 8 ml of additional titanium precursor was injected with the same manner at a rate of 0.3 ml min^{-1}, after the system was rapidly heated to 290 ℃ and held at the temperature for 10 minutes. The further post-treatment of TiO_2 nanobipyramids was the same procedure as that of TiO_2 nanoplates. Co-TiO_2 nanobipyramids were synthesized using the same approach as TiO_2 nanobipyramids, employing a mixed precursor solution of Co and Ti. A mixture of precursor solution was prepared by mixing the $Co(acac)_2$ stock solution and the titanium precursor solution (TiF_4/$TiCl_4$, 1/4) in a Co/Ti ratio of 3/8. The synthetic procedure was the same as that for Co-TiO_2 nanoplates.

Structural Characterizations X-ray diffraction patterns were acquired using an Empyrean X-Ray diffractometer with Cu Kα radiation (λ=1.5418Å). TEM images were obtained using FEI Spirit (120 kV). High-angle annular dark-field scanning TEM (HAADF-STEM) images were

captured using an Hitachi HD2700C dedicated STEM at an operating voltage of 200 kV, equipped Gatan Enfinium EELS spectrometer, at the Center for Functional Nanomaterials at Brookhaven National Lab. SEM-EDS analysis was performed using an FEI Quanta 650 microscope. Co K-edge EXAFS spectra were collected at 20BM of the Advanced Photon Source, Argonne National Laboratory, using a synchrotron X-ray source. The EXAFS data processing was conducted with standard methods with the Athena program. Least-squares curve fitting analysis of the EXAFS $\chi(k)$ data was performed using the ARTEMIS program. The model utilized for the analysis was built with substitution of central atom Ti in anatase phase TiO_2 with Co atom. Raman spectra were obtained with a Horiba LabRAM HR Evolution confocal Raman microscope.

Electrocatalytic measurement of OER All electrocatalytic performance assessment was conducted at room temperature in O_2 saturated 1M KOH electrolyte. Three electrode testing system comprised a carbon paper working electrode, a platinum wire counter electrode and a Hg/HgO (1M KOH) reference electrode. Bio Logic (Model VMP3) potential station was used to conduct all electrochemical tests. Co-TiO_2 and TiO_2 electrodes were prepared by airbrushing Co-TiO_2 and TiO_2 hexane dispersion on carbon paper, followed by annealing in air at 200 ℃ overnight to remove organic surfactant. Before electrocatalysis characterization, the reference electrode (Hg/HgO) was calibrated to reversible hydrogen electrode (RHE) via open circuit voltage test. The potential was reported vs. RHE potential according to the equation E (vs. RHE) = E (vs. HgO) + 0.926 V, where 0.926 V represents the potential difference between the reference electrode and RHE in the electrolyte (**Figure S34**). The overpotential at certain current density was calculated using the equation η = E (vs. RHE) - 1.23 V. In electrode activation process, the electrode was scanned with cyclic voltammetry test in the potential range of 0.776-0.926V vs. RHE for 500 cycles to remove extra fluorine and carbon on the electrode surface. Catalytic performance was

evaluated using linear sweep voltammetry (LSV) at a scan rate of 10 mV s^{-1} from 0.926 -1.676 V vs. RHE with IR compensation (85%). Catalytic stability test was carried out with chronoamperometry (CA) measurement under different potentials. The catalysts after CA test were collected from the carbon paper electrode by sonication and used for further post-catalysis characterization. To elucidate intrinsic activity of single-site Co on TiO_2, the turnover frequency (TOF) of catalysts was calculated by normalizing the activity to each Co atom. The electrochemical active surface area (ECSA) was determined with the equation, $ECSA = C_{dl}/C_s$, where C_{dl} is double layer capacitance in non-Faradic region and measured by cyclic voltammetry (CV) tests, and C_s is the specific capacitance of anatase Co-TiO_2 calculated from GCQM method under operando conditions (12 μF.cm^{-2}). The amount of Co sites in Co-TiO_2 nanoplates and nanobipyramids surface was estimated from ECSA and atom density in Co-TiO_2 surface and described by the following equation: $N_{\text{Co-atom}} = ECSA \times \frac{n \times w\%}{D_{cell}}$, where n is the number of Ti atoms in single cell of anatase TiO_2 surface of (001) and (101), w is atomic ratio of Co element in Co-TiO_2, and D_{cell} is the dimension of (001) and (101) facet in a single cell of anatase TiO_2. TOF was calculated by TOF = $\frac{j_{Geo} \times s}{4 \times N_{Co-atom} \times F}$, where j_{Geo} is geometric current density in LSV plot, s is electrode geometric area of working electrode, and F is Faraday constant.

Faradic efficiency (FE) towards OER was evaluated by a controlled electrolysis process in a gas-tight H-type cell which was separated by a Nafion-212 membrane and filled with N_2-saturated 1M KOH electrolyte in both compartments. A steady N_2 gas flow was fed into anode compartment during electrolysis with a rate of 10 sccm and the gas-phase effluent was continuously characterized with online gas chromatograph (Shimadzu, GC 2014). The concentration of O_2 was analyzed by a thermal conductivity detector and further used to calculate the FE.

Computational details

Our QM calculations used the Vienna ab initio simulation package (VASP ver. 5.4.5)[1-2] with the VASPsol solvation model[3] for geometry optimization followed by single point calculations as a function of applied potential using the CANDLE solvation model[4], as incorporated in the joint density-functional theory (JDFTx)[5].

Electron exchange and correlation were treated within the generalized gradient approximation (GGA)[6] using the PBE functional but including van der Waals attraction (D3 correction).[7] The interaction between the ionic core and the valence electrons was described by the projector-augmented wave (PAW) method.[8] Our model system employed 5 trilayers of 2 × 2 TiO_2 (001) and (101) slab with 15 Å of vacuum while the bottom three and two trilayers were fixed for (001) and (101) models, respectively.

We used a plane-wave basis set with an energy cutoff of 500 eV. The Brillouin zone was sampled using the 3x3x1 Monkhorst-Pack grid.[9] The convergence criteria for the electronic structure and the atomic geometry were 10^{-5} eV and 0.03 eV/Å.

Benchmark calculations were performed using the meta-GGA r2SCAN functional[10] for the water dissociation step (state 3 to state 4) on the Co-TiO_2 (001) surface. The reaction energy obtained with r^2SCAN is only 0.06 eV lower than the PBE-D3 value of 0.12 eV, supporting the reliability of our calculations.

The JDFTx calculations used the GBRV[11] ultrasoft pseudopotential (USPP) with a plane wave cutoff of 544 eV (20 Hartree). The ionic screening of different net charges was achieved with 0.1 M K+ and 0.1 M F- in the fluid model. All other settings are similar to those in VASP calculations.

The Gibbs free energy is calculated at 298 K and 1 atm as:

$$G = H - T\Delta S = E_{DFT} + E_{ZPE} + E_{solv} + \int_0^{298} C_v dT - T\Delta S$$

where E_{DFT} is the electronic total energy, E_{ZPE} is zero-point vibrational energy, and E_{solv} is the solvation energy. The enthalpy ($\int_0^{298} C_v dT$) and entropy (ΔS) contributions to G were calculated at room temperature.

Grand canonical free energies were obtained for all states using the grand canonical potential (GCP) method[11] to determine the effect of surface charge on the adsorbates at constant potential.

Summary of the GCP-K Formulation In this study, the grand canonical free energies were obtained for all states using the GCP-K method[12] to determine the effect of surface charge on the adsorbates at constant potential. Normally, QM calculations are carried out for a fixed number of electrons, whereas electrocatalysis is carried out under conditions of a fixed applied potential. To modify QM to describe constant potential requires a complex correction obtained by determining the relationship between the work function of the slab surface and the number of electrons.[13-15] Instead we apply the Legendre transformation (eq. 1) to obtain the grand canonical potential (GCP).

The GCP is derived from a combined solvent-slab free energy including the counterion charge distribution in the implicit solvation model using generalized Poisson-Boltzmann equation[1–3] given by

$$G(n;u) = F(n) - ne(U_{SHE} - U) \qquad (1)$$

where G is the grand canonical free energy, which depends on the applied potential (U) and on the number of electrons n. Here, e is the unit charge, F is the total free energy as a function of n, and U_{SHE} is the standard hydrogen electrode (SHE) potential. The signs are chosen to compare results directly with experiment, i.e., $U = +1.0$ V in experiment corresponds to +1.0 V vs SHE. To change

to the reversible hydrogen electrode (RHE), we shift the reference Fermi level, which depends on the pH of the solution. As expected from the solutions to Poisson's equation, *F(n)* has a quadratic form given by

$$F(n) = a(n - n_0)^2 + b(n - n_0) + c \quad (2)$$

where the *a, b*, and *c* parameters are fitted according to the QM calculations. Here *a* should be positive to obtain a stable system and n_0 is the number of valence electrons for a neutral system. The quadratic form of free energy is strictly verified in calculations. With the quadratic equation (eq. 2), we selected valid points relevant to the OER working condition to calculate the GCP via Legendre transformation, ensuring that the energetics were derived based on interpolation of data points from DFT calculations (not extrapolation). We adjust the number of electrons to match the electronic Fermi level to the applied potential in the constant potential condition, as described in eq. 3.[4, 5]

$$\frac{dG(n;U)}{dn} = 0 \text{ or } \mu_e = \frac{dF(n)}{dn} = e(U_{SHE} - U) \qquad (3)$$

The minimization leads to the GCP that matches the applied potential. This is defined in eq. 4. The coefficients are very important because it determines the speed at which the GCP changes with the applied potential. GCP(U) is linearly related to n_0, leading to a single intersection between n_0 with U. Thus, a structure with greater total charge (larger n_0) will lead to a faster change in the GCP with applied potential.

$$\mathrm{GCP(U)} = \min_n G(n;U) = \min_n (F(n) - ne(U_{SHE} - U)) = -\frac{1}{4a}(b - \mu_{e,SHE} + eU)^2 + c - n_0 \mu_{e,SHE} + n_0 eU \qquad (4)$$

Using this form, we relate the parameters *a, b*, and *c* to physical quantities as follows:

• The free energy $F(n_0) = c$ when the system is neutral.

• The differential capacitance is $C_{diff} = \frac{\partial n}{\partial U} = -\frac{1}{2a}$.

• The number of electrons is $n(U) = -\frac{1}{e}\frac{\partial GCP(U)}{\partial U} = n_0 - \frac{1}{2ae}(b - \mu_{e,SHE} + eU)$. So, $b = \mu_{e,SHE} - e U_{PZC}$, where U_{PZC} is the potential of zero charge.

Summarizing, the grand canonical potential and the free energy have the following form in terms of physical quantities:

$$n = n_0 + C_{diff}(U - U_{PZC}) \quad (5)$$

$$GCP(U) = \frac{e^2 C_{diff}}{2}(U - U_{PZC})^2 + n_0 eU + F_0 - n_0 \mu_{e,SHE} \quad (6)$$

$$F(n) = -\frac{1}{2C_{diff}}(n - n_0)^2 + (\mu_{e,SHE} - eU_{PZC})(n - n_0) + F_0 \qquad (7)$$

When the GCP is at its minimum, there is an inverse relationship between the number of charges and applied potential (C_{diff} has a positive value), which means that the electron charge will increase if a negative potential is applied. Since the second derivative of GCP(U) with respect to potential is small and the minimum potential value of the grand canonical potential is far from zero potential, we can use the first-order expansion at zero potential to approximate GCP(U) (eq 5), leading to a nearly straight line, as reported previously.[7, 8] Our calculations confirm that non-magnetic TiO_2 as the host material results in minimal magnetic degrees of freedom, with negligible energy differences due to spin degeneracy within the relevant potential range. These results validate the robustness of our methodology and the quadratic dependence of free energy, as shown in **Figure S33**.

Method to calculate Co atomic area density The density (D_{Area}) of cobalt atoms normalized on area in nanobipyramid and nanoplate is calculated according to following equation:

$$D_{Area} = (\frac{4}{D_{Cell\,(001)}} \times R_{(001)} + \frac{12}{D_{Cell\,(101)}} \times R_{(101)}) \times w\%$$

where 4 is the number of Ti atoms in single cell of anatase TiO_2 surface of (001), 12 is the number of Ti atoms in single cell of (101); $D_{Cell\ (001)}$ is the dimension of (001) in a single cell, 0.6 nm^{-2}; $D_{Cell\ (101)}$ is the dimension of (101) in a single cell, 1.16 nm^{-2}; $R_{(001)}$ is geometrical area ratio of (001) facet; $R_{(101)}$ is geometrical area ratio of (101) facet; w is w is atomic ratio of Co element in Co-TiO_2.

Calculation of area and ratio of facets in antase TiO_2 nanocrystals The area of facet {001} and {101} was calculated based on the ideal model of truncated tetragonal bipyramid TiO_2 with selectively facet exposure of {001} and {101}, as shown in Fig. S15 with atomic model illustration. The calculation of area of {001}and {101} facets was according to following equations:

$$A_{001} = 2 \times (w - l \times (a/c))^2$$

$$A_{\{101\}} = 2 \times l^2 \times (2 \times (w/l) - a/c) \times \sqrt{1 + (a/c)^2}$$

where l is length along a axis, w is width along c axis, a and c are lattice constants with a 0.379 nm, c 0.951 nm, as illustrated in Fig. S20. The results were listed in Table S1.

Supplementary Table

Table S1. Structural parameters of TiO_2 and Co-TiO_2 nanobipyramids/nanoplates obtained from Ti K-edge and Co K-edge EXAFS spectra.

	Sample	Path	CN	R (Å)	ΔE (eV)	σ^2 (10^{-3} Å)	R-factor
Co K-edge	Co-TiO_2 nanobipyramids	Co-O	4.67±0.31	2.085±0.007	-1.00±0.68	0.0069±0.0011	0.002
	Co-TiO_2 nanoplates	Co-O	4.47±0.31	2.048±0.009	-1.85±0.73	0.0063±0.0014	0.005
	Co-TiO_2 nanobipyramids after EC	Co-O	4.51±0.48	2.034±0.011	-1.59±1.23	0.0099±0.0019	0.007
	Co-TiO_2 nanoplates after EC	Co-O	4.43±0.50	2.021±0.011	-3.17±1.19	0.0073±0.0018	0.003
Ti K-edge	Co-TiO_2	Ti-O	6	1.950±0.013	-2.59±1.77	0.0032±0.0017	0.007
	nanobipyramids	Ti-Ti/Co	4	2.988±0.022	8.19±6.08	0.0025±0.0014	0.0108
	Co-TiO_2 nanoplates	Ti-O	6	1.949±0.022	-2.18±3.14	0.0025±0.0029	0.0124
		Ti-Ti/Co	4	2.993±0.039	-12.51±5.91	0.0026±0.0026	0.0133
	Co-TiO_2 nanobipyramids after EC	Ti-O	6	1.952±0.010	-2.12±1.42	0.0027±0.0013	0.009
		Ti-Ti/Co	4	3.003±0.016	-11.65±2.50	0.0025±0.0011	0.014
	Co-TiO_2	Ti-O	6	1.954±0.010	-2.43±1.25	0.0066±0.0015	0.006
	nanoplates after EC	Ti-Ti/Co	4	2.997±0.019	-13.27±2.38	0.0063±0.0012	0.019
	TiO_2 nanobipyramids	Ti-O	6	1.947±0.013	-2.59±1.77	0.0031±0.0017	0.007
		Ti-Ti/Co	4	2.992±0.020	-12.72±3.08	0.0024±0.0014	0.011
	TiO_2 nanoplates	Ti-O	6	1.949±0.022	-2.18±3.14	0.0025±0.0029	0.0123
		Ti-Ti/Co	4	2.993±0.039	-12.51±5.91	0.0026±0.0026	0.0172

Table S2. Area and facet ratio of {001} and {101} in Co-TiO_2 nanocrystals.

Sample	l (nm)	w (nm)	$A_{(001)}$ (nm^2)	$A_{(101)}$ (nm^2)	$R_{\{001\}}$ (%)	$R_{\{101\}}$ (%)
**Nanoplates**	**5.8**	**16.7**	**414.1**	**388.2**	**51.6**	**48.4**
**Nanobipyramid**	**17.0**	**9.3**	**12.8**	**432.8**	**2.9**	**97.1**

Table S3. Co-based electrocatalyst performance comparison with the reported results under the similar conditions.

Catalyst	Overpotential (mV)	Electrolyte	TOF (s^{-1})	TOF calculation method	Ref
Hollow $LaCoO_3$ nanospheres	370	0.1 M KOH	8.0×10^{-3}	Calculated based on the total current (or detected O_2), and mole of Co atoms[15-18], or total moles of metals[16].	16
	415		2.6×10^{-2}		
Co-rGO	300		6.03		17
NiCo-rGO			7.34		
CoFe-rGO			1.68		
Nitrogen-Doped Mesostructured Carbon-Supported Metallic Cobalt Nanoparticles	400		0.086		18
Electrodeposited Cobalt phosphate	410	0.1 M phosphate buffer (pH = 8)	2×10^{-3}		19
Monolayer Co NP assembly	400	0.1 M KOH	2.13	Calculated by the total current or the surface Co or Co + Fe atom amounts are estimated using electrochemical approaches such as interrogation scanning electrochemical microscope (SI-SECM) analysis[20] or using physical characterization approaches such as surface TEM analysis[19] or with unit cell model analysis[21].	22
Co-PPy	350		0.32		21
CoFe-PPy			0.45		
$NiFeO_xH_y$ particles (surface)	300		6.2 ± 1.6		20
$FeCoS_2$/XC-72	230		1.428	Calculated by the total current and the mole of active sites (Co). The method to determine active site amount was not reported	23
Hetero-N-Coordinated Co Single Sites	265	0.5 M H_2SO_4	2.8		24
Co_3C-NB (NB-doped)	354	phosphate buffer (pH = 7)	0.05		25
Co_3C-B	449				
Co_3C-N	460				
Co_3C	487				

Table S4. Gibbs free energy of different surface states on TiO_2 (001) and (101) surfaces.

Energy [eV]	H_2O^* 1 ML	*OH 1 ML	*O 1 ML

TiO_2 (001)	-587.27	-565.984	-543.205
TiO_2 (101)	-694.485	-670.679	-647.614

Supplementary Figures

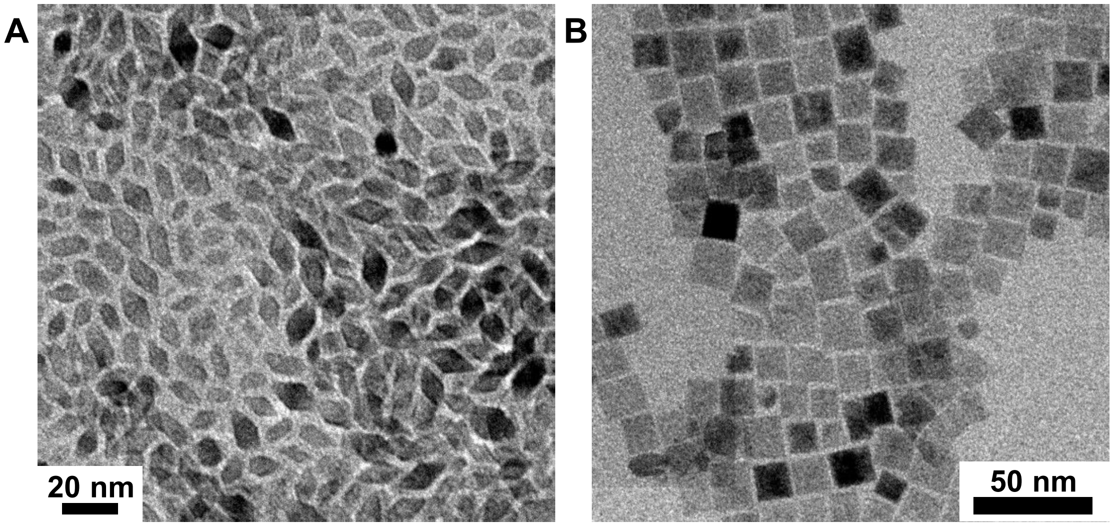

Figure S1. TEM images of TiO_2 nanocrystals. (A) TiO_2 nanobipyramids. (B) TiO_2 nanoplates.

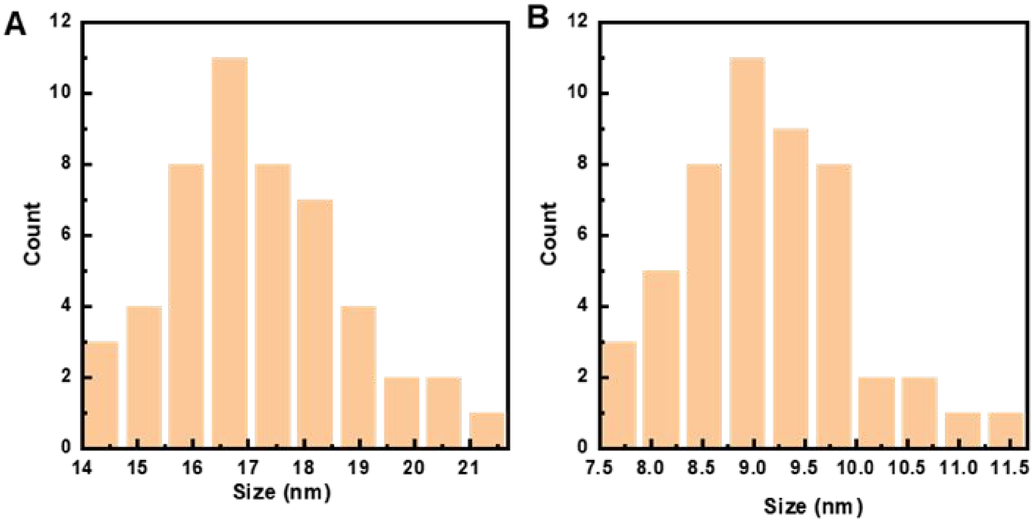

Figure S2. Size distribution analysis of Co-TiO_2 nanobipyramids. (A) Size distribution in length along c axis. (B) Size distribution in width along a axis. (a and c axes are illustrated in **Figure S20**)

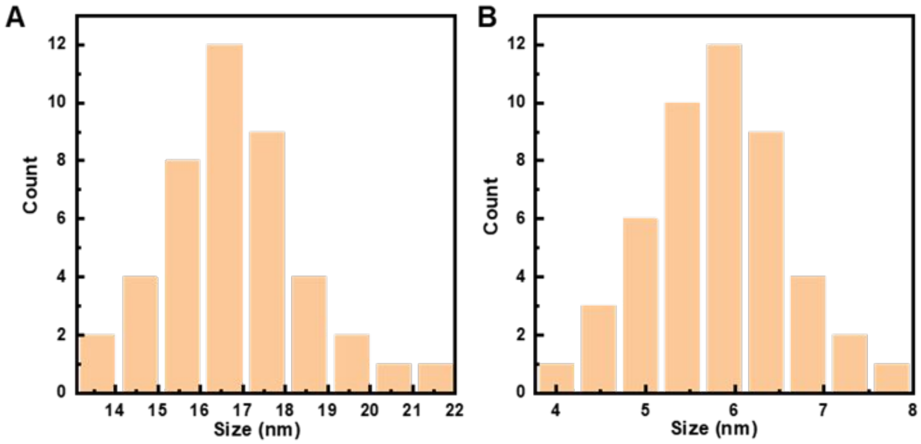

Figure S3. Size distribution analysis of Co-TiO_2 nanoplates. (A) Size distribution in width along a axis. (B) Size distribution in length along c axis. (a and c axes are illustrated in **Figure S20**)

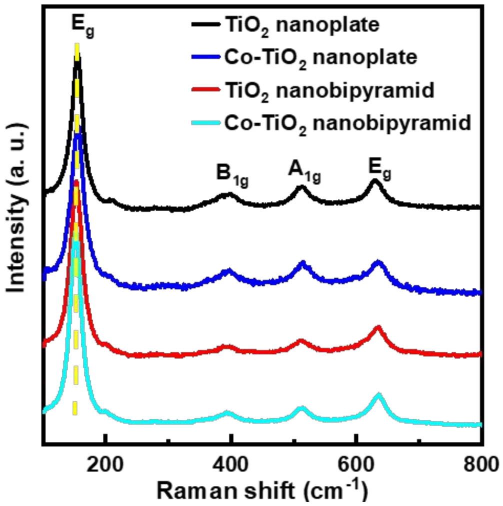

Figure S4. Raman spectra in the range of 100-800 cm^{-1} for the TiO_2 nanoplate, Co-TiO_2 nanoplate, TiO_2 nanobipyramid, and Co-TiO_2 nanobipyramid samples.

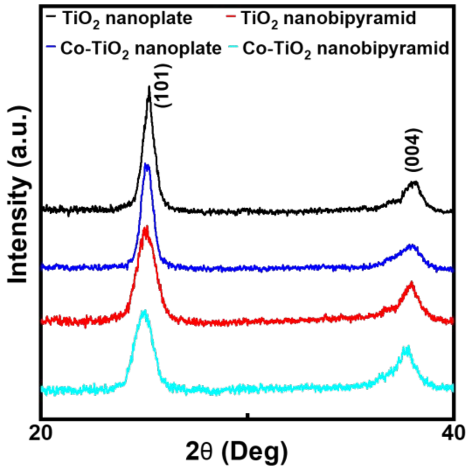

Figure S5. Zoom-in view of XRD patterns of TiO_2 and Co-TiO_2 nanocrystals with indexed peaks.

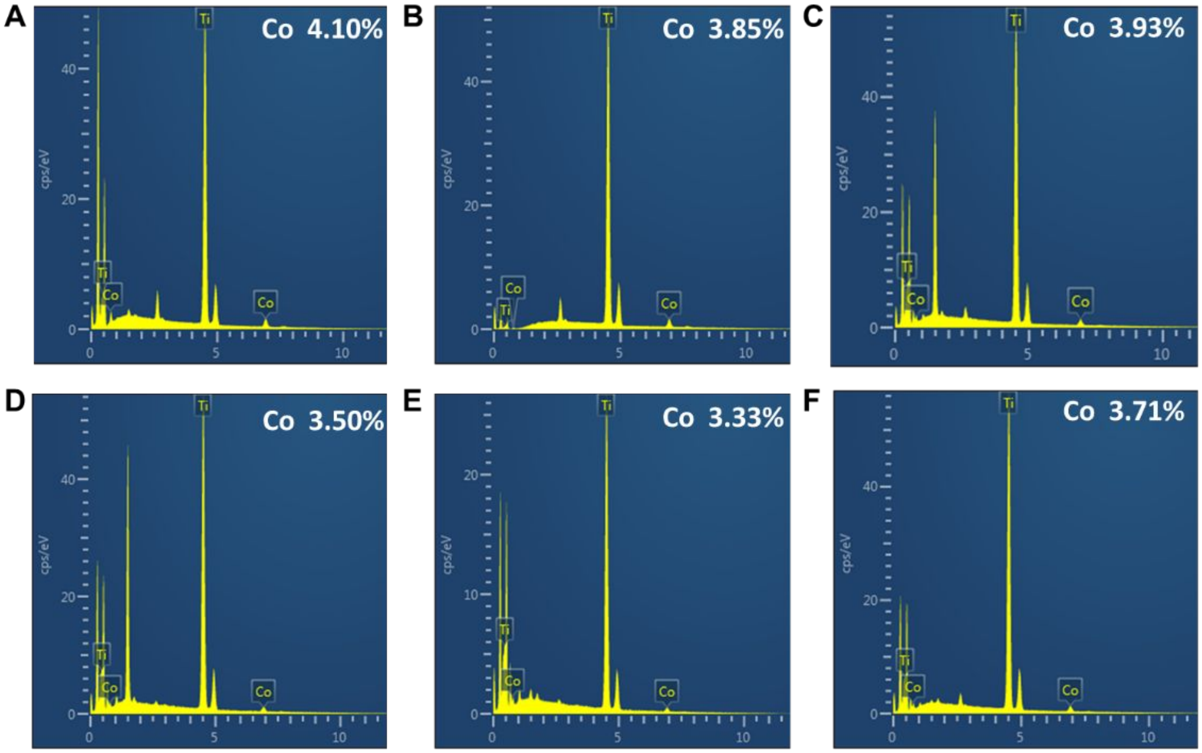

Figure S6. EDS spectra of Co-TiO_2 nanocrystals: (A-C) for Co-TiO_2 nanobipyramids. (D-F) for Co-TiO_2 nanoplates.

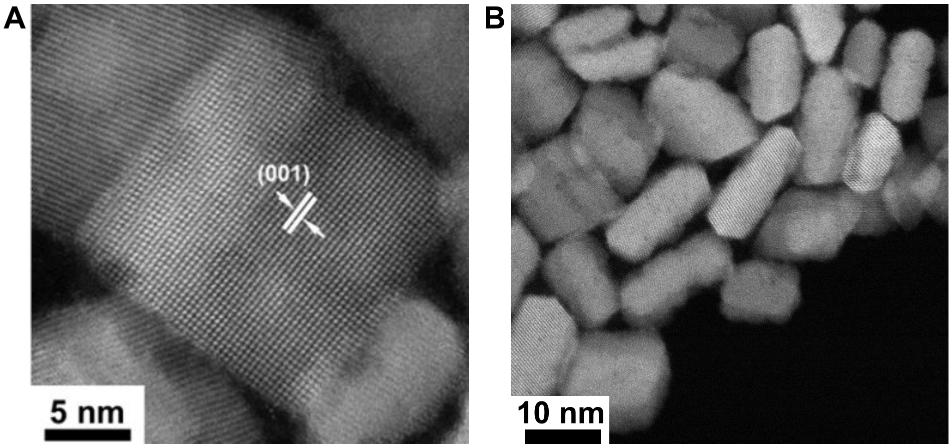

Figure S7. STEM image of Co-TiO_2 nanoplate and the cross-section image.

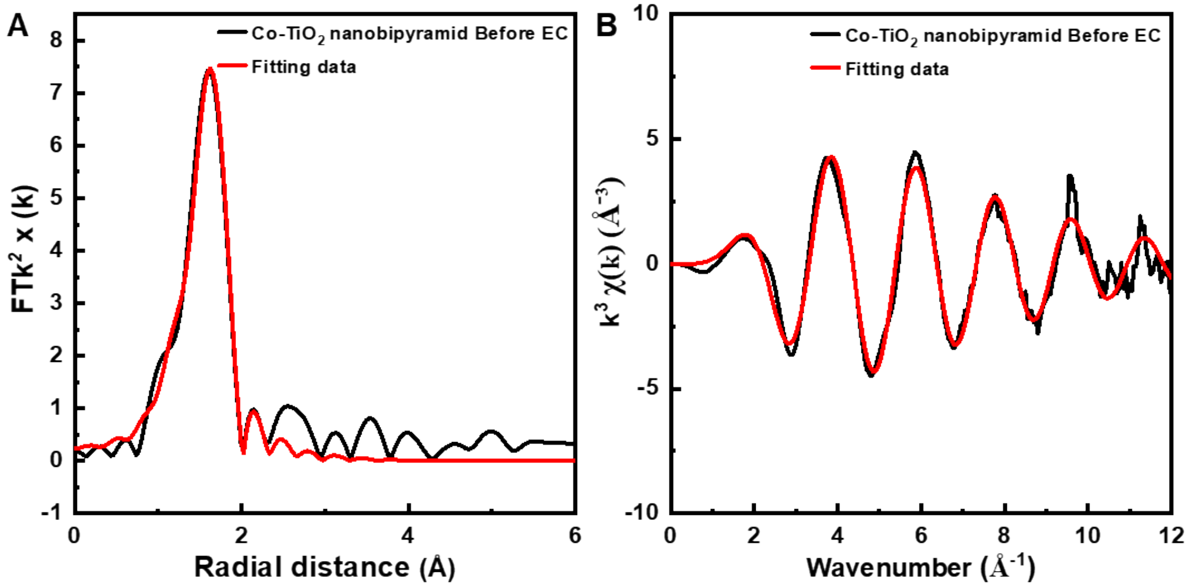

Figure S8. Co K-edge EXAFS spectra fittings for Co-TiO_2 nanobipyramids. (A) Fitting in R-space. (B) Fitting in k-space.

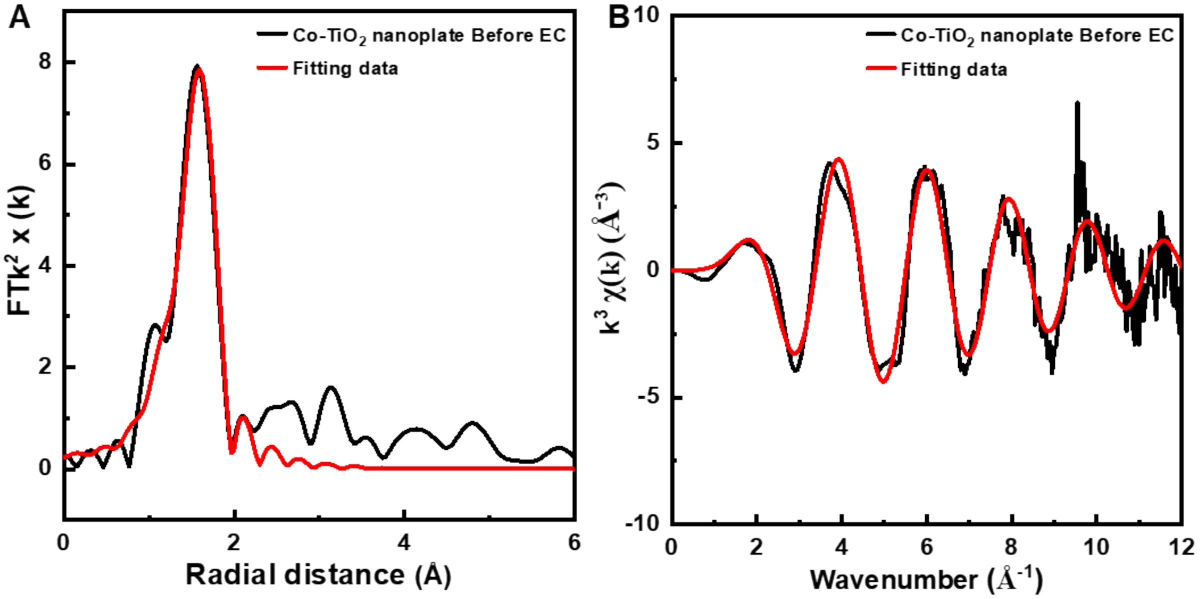

Figure S9. Co K-edge EXAFS spectra fittings for Co-TiO_2 nanoplates. (A) Fitting in R-space. (B) Fitting in k-space.

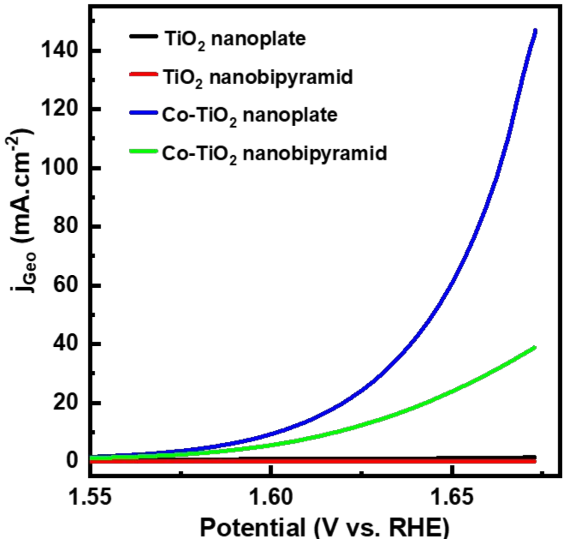

Figure S10. Zoom-in view of LSV plots for different catalysts.

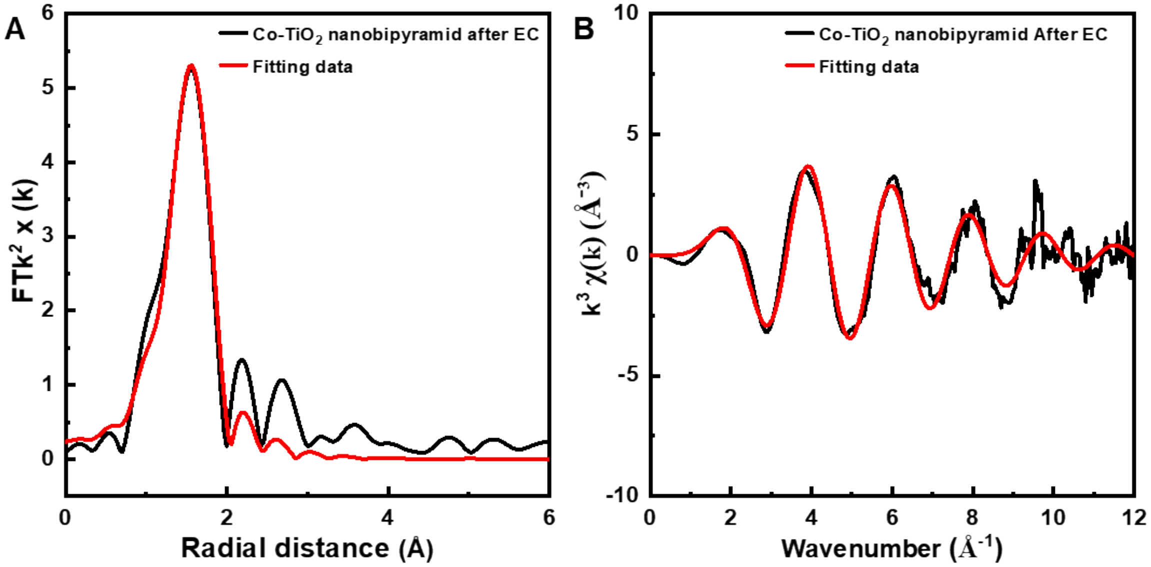

Figure S11. Co K-edge EXAFS spectra fittings for Co-TiO_2 nanobipyramids after electrochemical test. (A) Fitting in R-space. (B) Fitting in k-space.

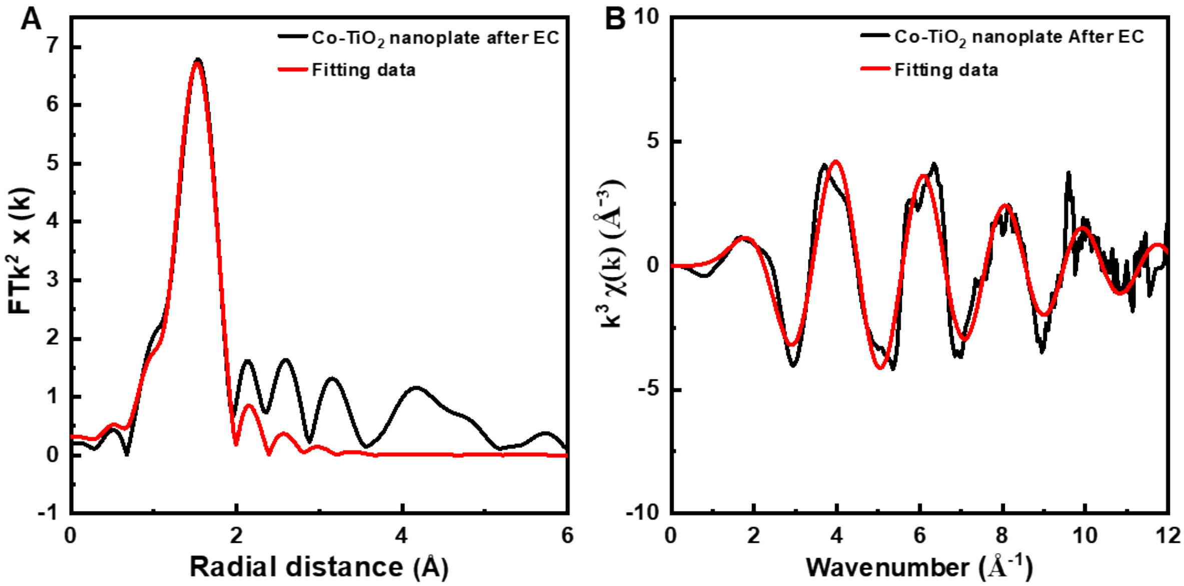

Figure S12. Co K-edge EXAFS spectra fittings for Co-TiO_2 nanoplates after electrochemical test. (A) Fitting in R-space. (B) Fitting in k-space.

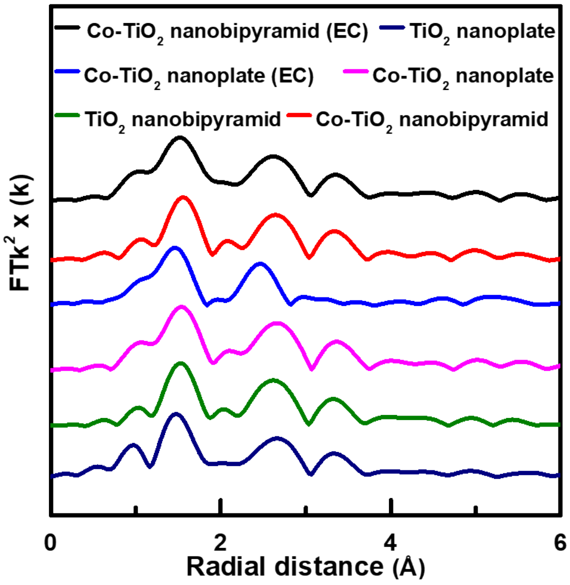

Figure S13. Ti K-edge EXAFS spectra of TiO_2 and Co-TiO_2 nanobipyramids and nanoplates before and after electrochemical test.

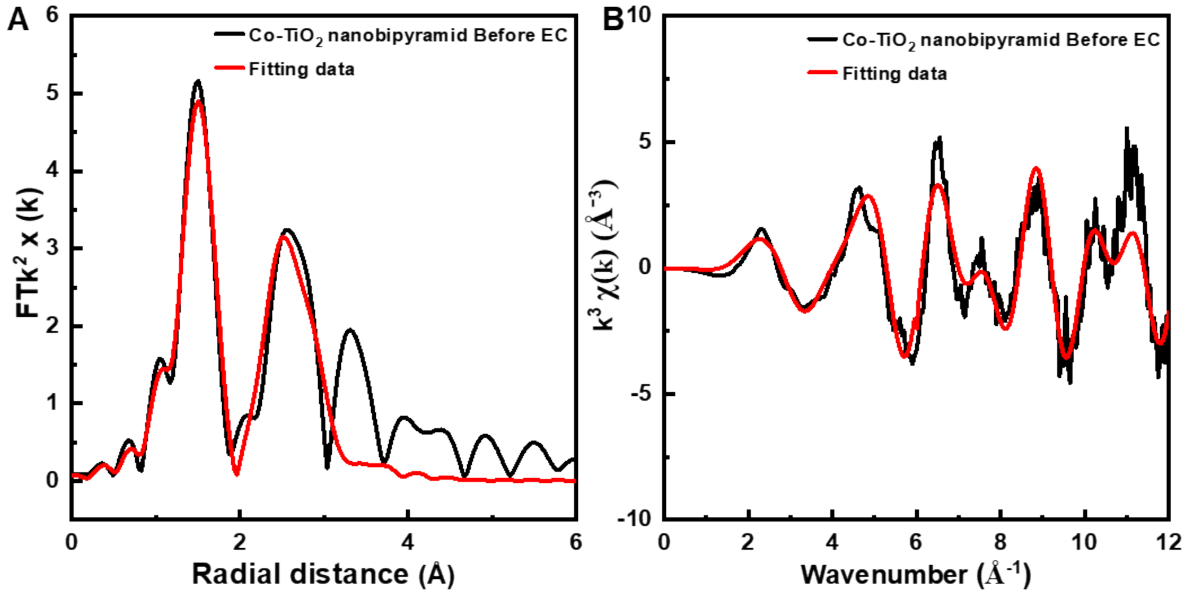

Figure S14. Ti K-edge EXAFS spectra fittings for Co-TiO_2 nanobipyramids. (A) Fitting in R-space. (B) Fitting in k-space.

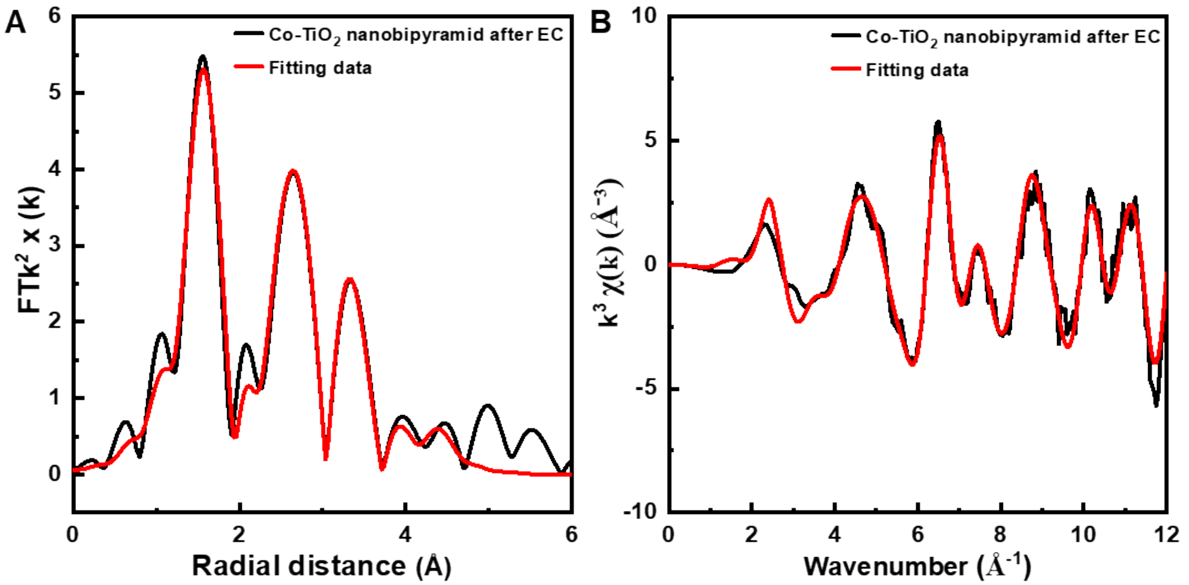

Figure S15. Ti K-edge EXAFS spectra fittings for Co-TiO_2 nanobipyramids after electrochemical test. (A) Fitting in R-space. (B) Fitting in k-space.

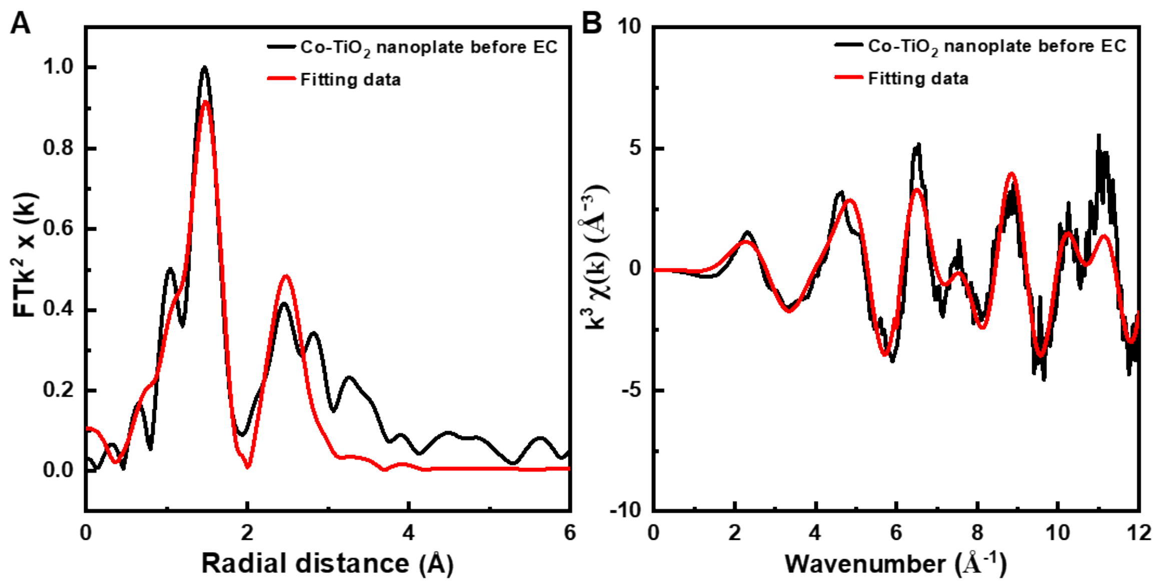

Figure S16. Ti K-edge EXAFS spectra fittings for Co-TiO_2 nanoplates. (A) Fitting in R-space. (B) Fitting in k-space.

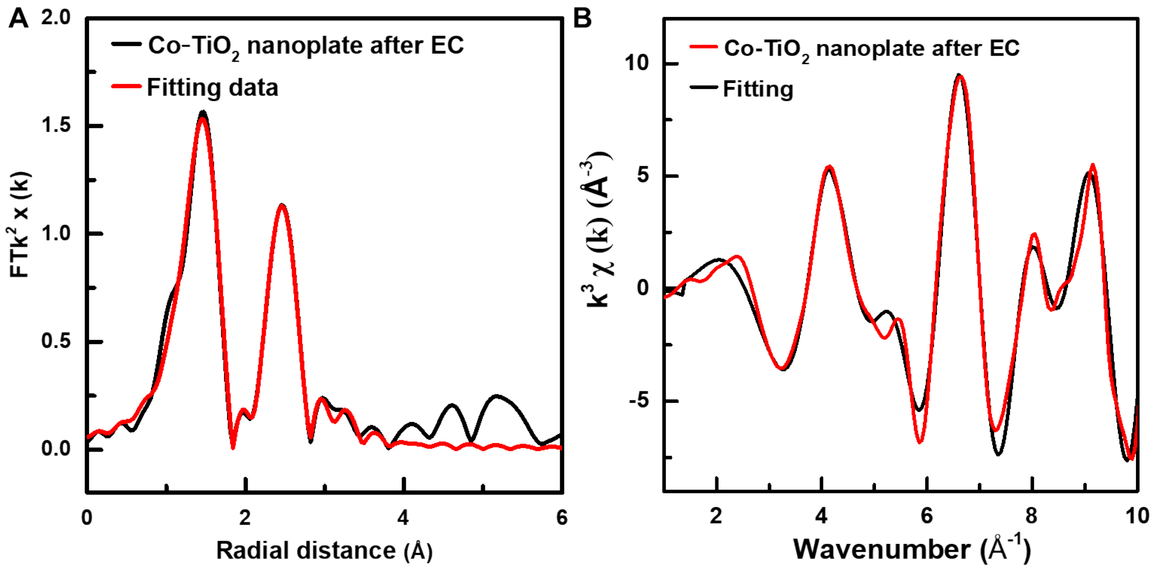

Figure S17. Ti K-edge EXAFS spectra fittings for Co-TiO_2 nanoplates after electrochemical test. (A) Fitting in R-space. (B) Fitting in k-space.

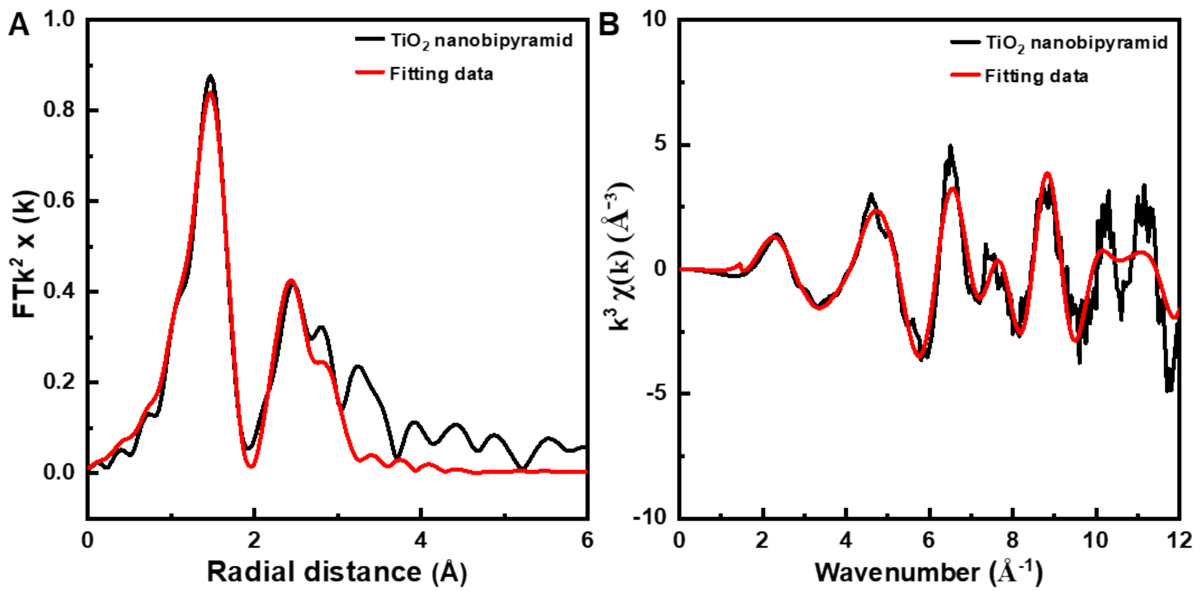

Figure S18. Ti K-edge EXAFS spectra fittings for TiO_2 nanobipyramids. (A) Fitting in R-space. (B) Fitting in k-space.

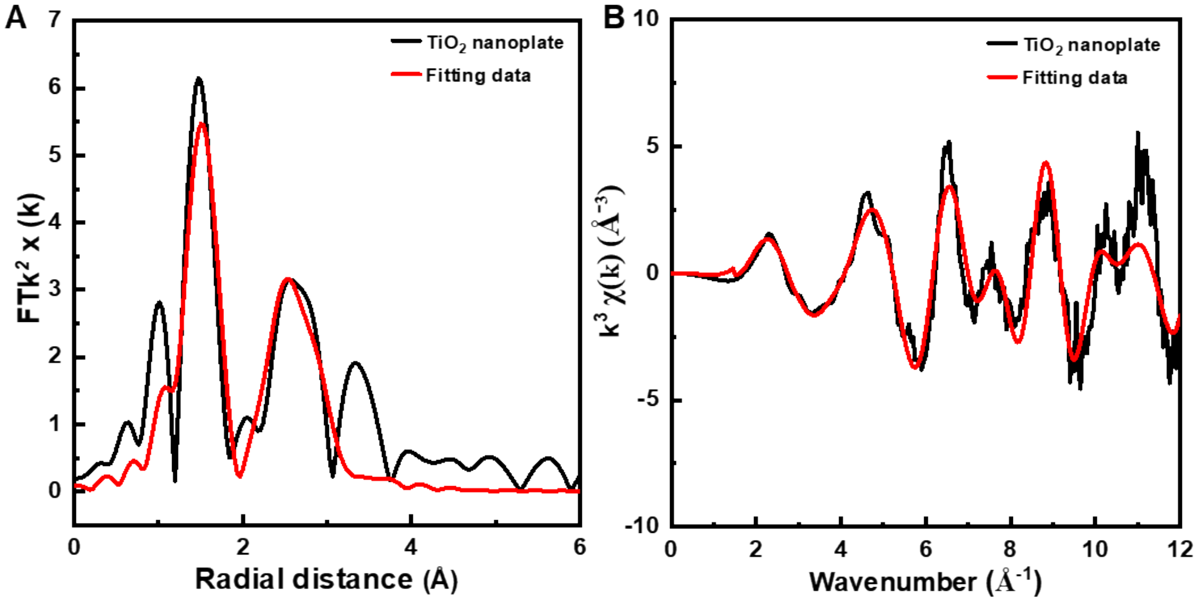

Figure S19. Ti K-edge EXAFS spectra fittings for TiO_2 nanoplates. (A) Fitting in R-space. (B) Fitting in k-space.

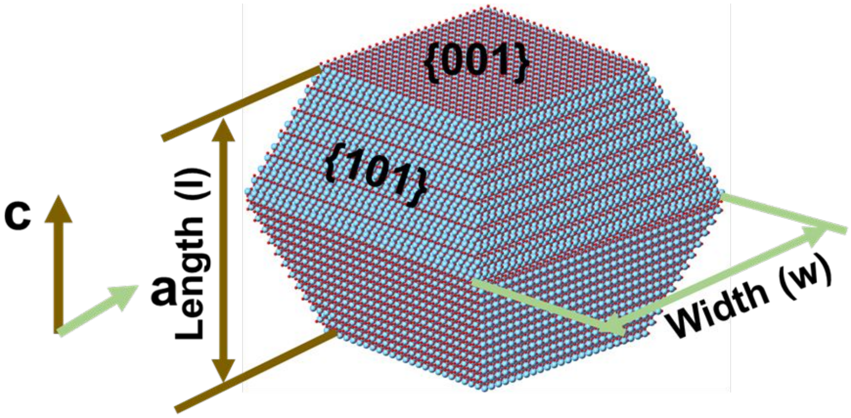

Figure S20. Atomic model illustrations of Co-TiO_2 truncated bipyramid with only exposure of {001} and {101} facets, with red and blue circles being oxygen and titanium.

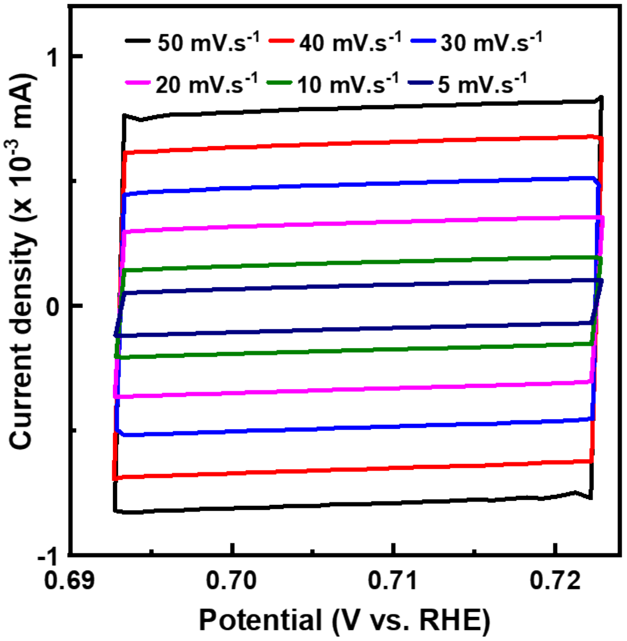

Figure S21. Cyclic voltammetry plots of Co-TiO_2 nanobipyramids at different scanning rates in the potential window of 0.692 V - 0.722 V vs. RHE.

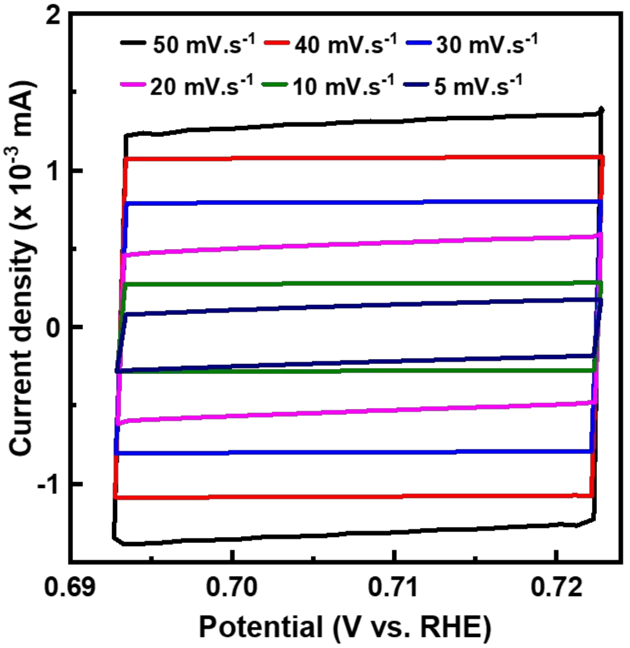

Figure S22. Cyclic voltammetry plots of Co-TiO_2 nanoplates at different scanning rates in the potential window of 0.692 V - 0.722 V vs. RHE.

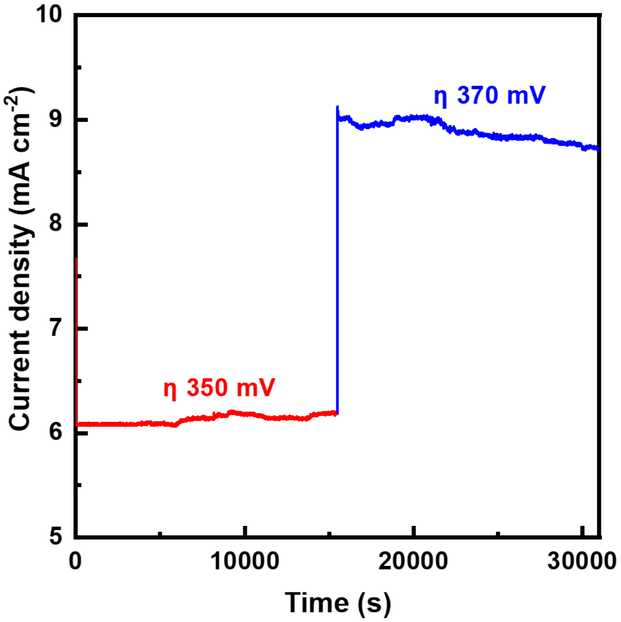

Figure S23. Chronoamperometry test of Co-TiO_2 nanoplates at different overpotential.

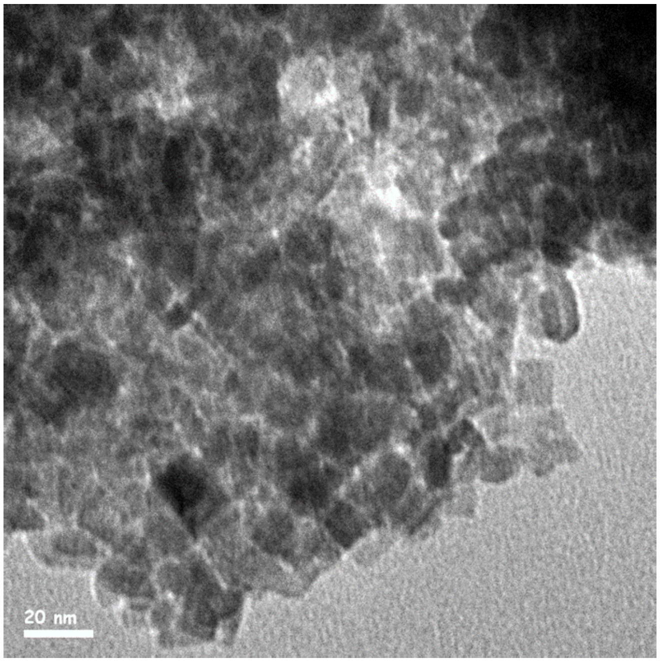

Figure S24. TEM image of Co-TiO_2 nanoplates after electrochemical test.

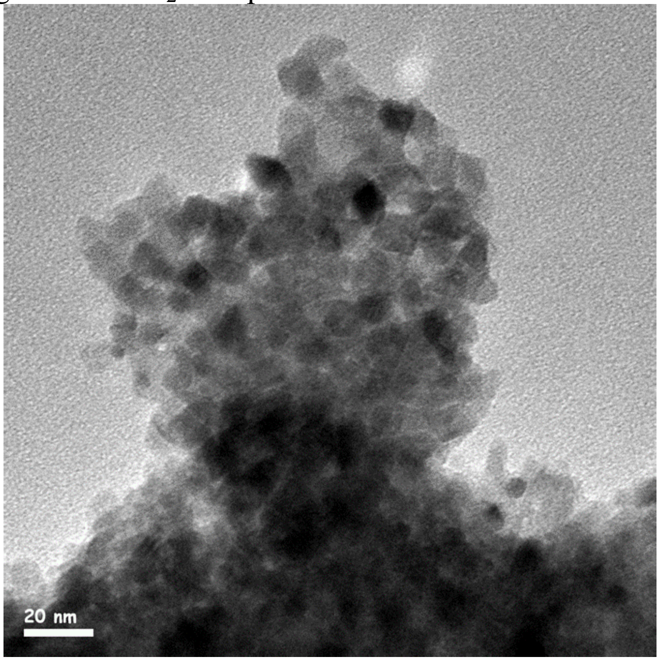

Figure S25. TEM image of Co-TiO_2 nanobipyramids after electrochemical test.

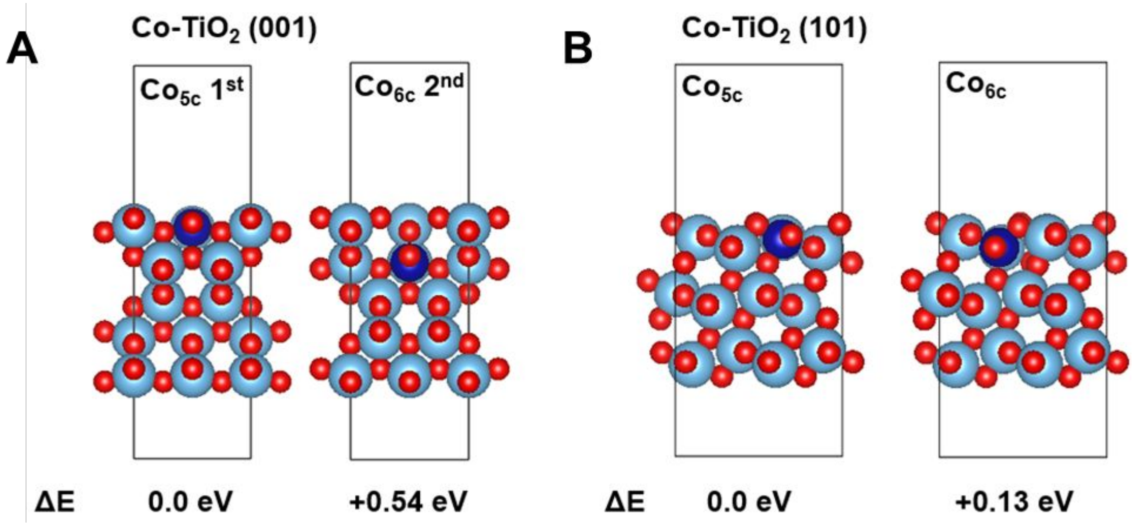

Figure S26. Slab models of the Co-doped Anatase TiO_2 structure. Blue, light blue, and red circles denote Co, Ti, and O atom respectively. (A**)** Side view of Co-TiO_2 (001). The Co substitution prefers Co_{5c} site in the 1st top layer by 0.54 eV to Co_{6c} in the sublayer. (B) Side view of Co-TiO_2 (101). The Co substitution prefers Co_{5c} site by 0.13 eV to Co_{6c} in the 1st top layer.

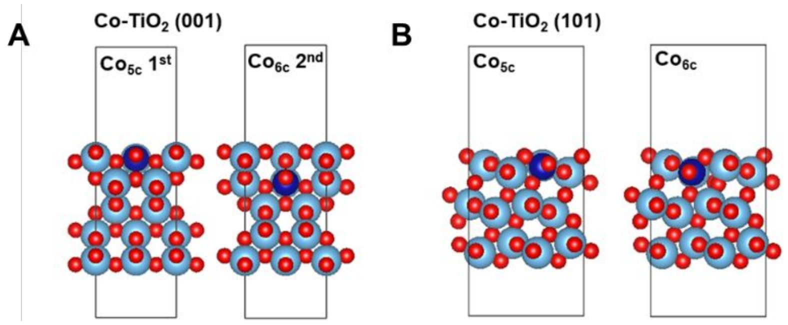

Figure S27. Schemes of Co substitution on different anatase TiO_2 facets.

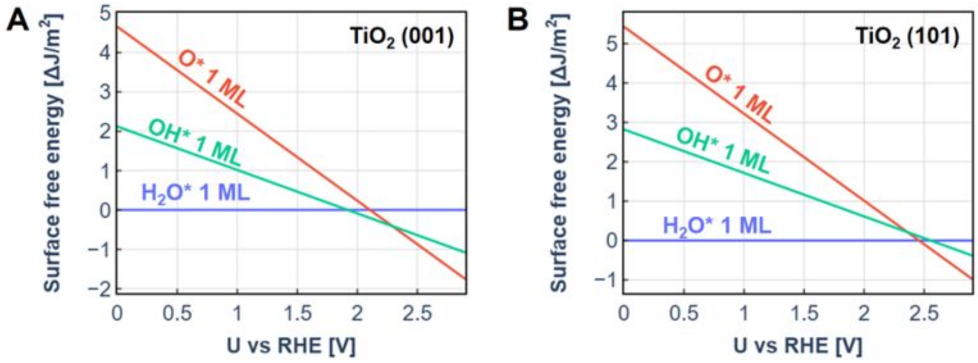

Figure S28. Surface energy diagram of (A) TiO_2 (001) and (B) TiO_2(101) facets as a function of applied potential.

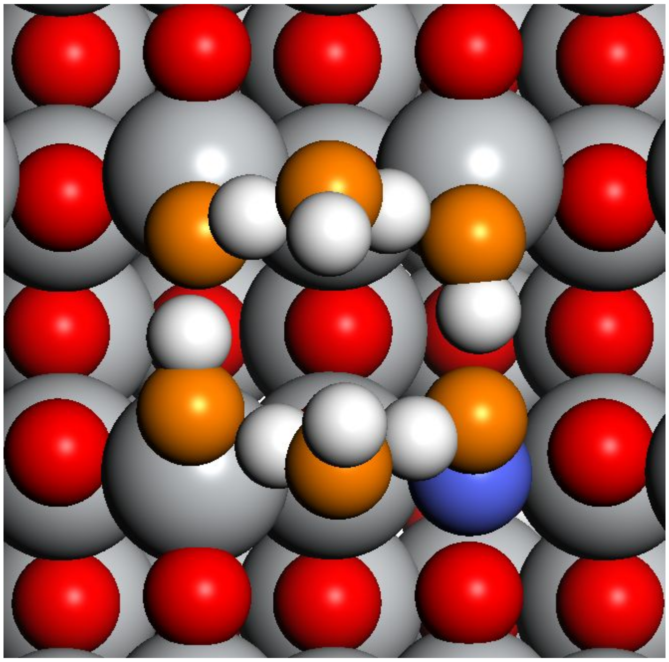

Figure S29. Water dissociated structure. The two water molecules among the four explicit H_2O (corresponds to one water monolayer) dissociatively adsorb on Co-TiO_2 (001) forming 4 surface hydroxides. Each color represents a different element: blue (Co), grey (Ti), white (H), red (lattice O), orange (O of the surface water).

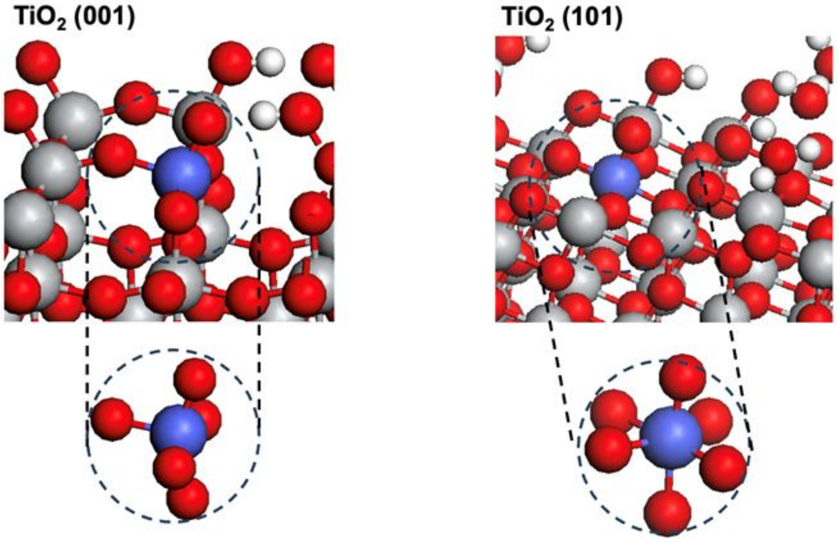

Figure S30. Reactive motifs on Co-TiO_2 (001) and (101) surfaces at their highest oxidation state (state 3) during the OER cycle.

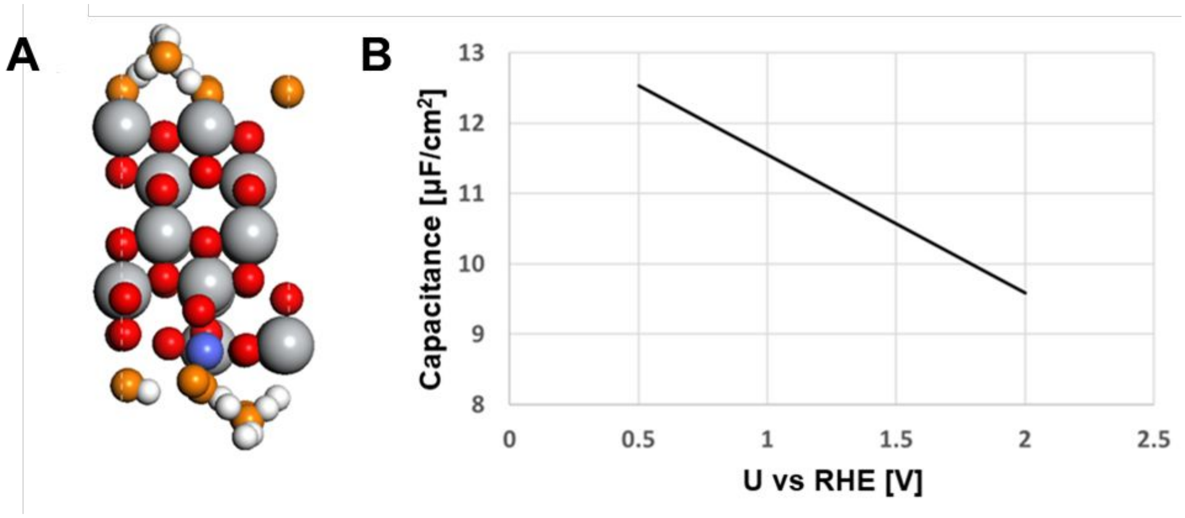

Figure S31. (A) A stable water dissociated structure with inversion symmetry. (B) The capacitance vs voltage plot which leads to 12.1 μF cm^{-2} at 0.69 < U < 0.72 V.

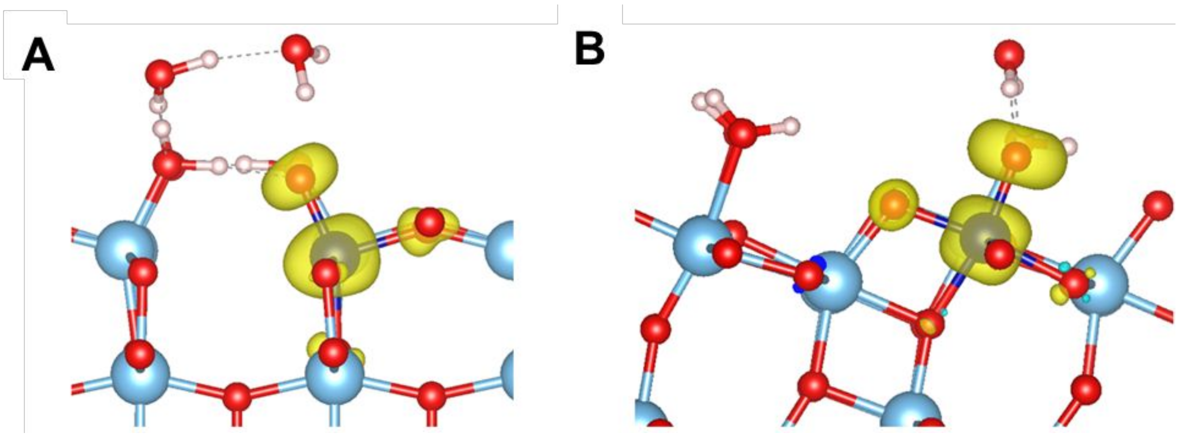

Figure S32. The spin density plots which show the net difference of up and down spin of state 3 (A) at U = 1.56 V for (001) surface and (B) at U = 1.45 V for (101) surface. The Bader charge analysis on (001) surface shows that the spin population leads to 2.1 e^- on Co and 0.77 e^- on the terminal oxo (=O_t) which is predicted to be a radical. The same analysis on (101) surface leads to 1.1 e^- on Co and 0.83 e^- on the =O_t.

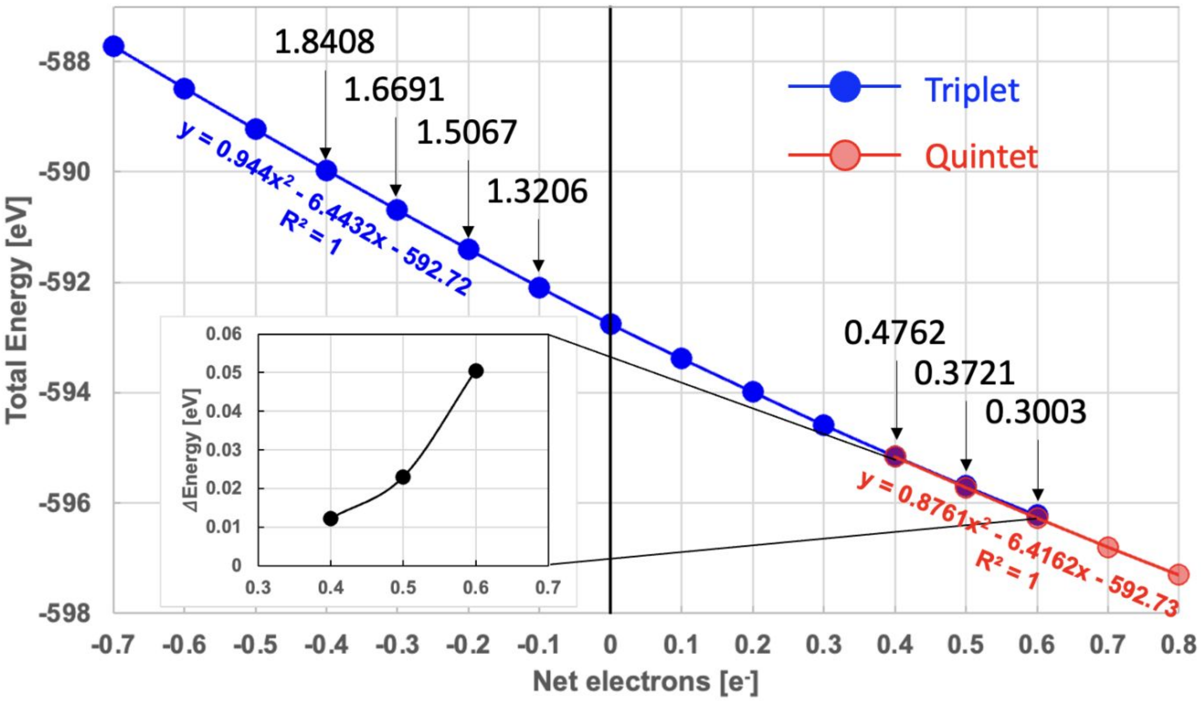

Figure S33. Total energy of two different spin states of state1 of Co-TiO_2 (001) surface with different number of net electrons. Each trendline is provided with 2nd order polynomial equation and R-squared value. The number with arrow indicates the applied voltage in RHE scale. The inset figure shows the energy difference of the triplet state from the quintet state.

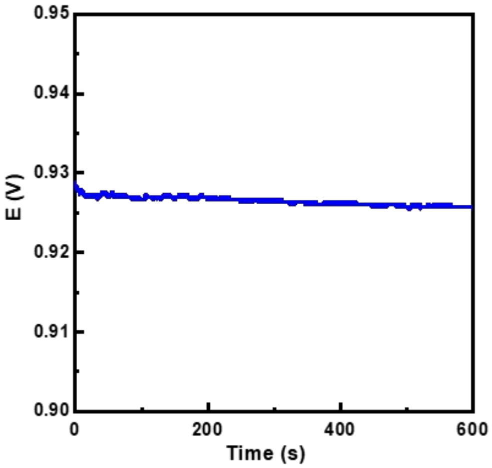

Figure S34. OCP measurement of Hg/HgO electrode vs. RHE.

Reference

1. Kresse, G.; Hafner, J. Ab initio molecular-dynamics simulation of the liquid-metal--amorphous-semiconductor transition in germanium. *Phy. Rev. B* **1994,** *49*, 14251-14269.
2. Kresse, G.; Furthmüller, J. Efficient iterative schemes for ab initio total-energy calculations using a plane-wave basis set. *Phy. Rev. B* **1996,** *54*, 11169-11186.
3. Mathew, K.; Sundararaman, R.; Letchworth-Weaver, K.; Arias, T. A.; Hennig, R. G. Implicit solvation model for density-functional study of nanocrystal surfaces and reaction pathways. *J. Chem. Phys.* **2014,** *140*, 084106.
4. Sundararaman, R.; Goddard, W. A., III. The charge-asymmetric nonlocally determined local-electric (CANDLE) solvation model. *J. Chem. Phys.* **2015,** *142*, 064107.
5. Sundararaman, R.; Letchworth-Weaver, K.; Schwarz, K. A.; Gunceler, D.; Ozhabes, Y.; Arias, T. A. JDFTx: Software for joint density-functional theory. *SoftwareX* **2017,** *6*, 278-284.
6. Perdew, J. P.; Burke, K.; Ernzerhof, M. Generalized gradient approximation made simple. *Phys. Rev. Lett.* **1996,** *77*, 3865-3868.
7. Johnson, E. R.; Becke, A. D. A post-Hartree-Fock model of intermolecular interactions: Inclusion of higher-order corrections. *J. Chem. Phys.* **2006,** *124*, 174104.
8. Blöchl, P. E. Projector augmented-wave method. *Phys. Rev. B* **1994,** *50*, 17953-17979.
9. Monkhorst, H. J.; Pack, J. D. Special points for Brillouin-zone integrations. *Phys. Rev. B* **1976,** *13*, 5188-5192.
10. Furness, J. W.; Kaplan, A. D.; Ning. J.; Perdew, J. P.; Sun, J. Accurate and numerically efficient r^2SCAN meta-generalized gradient approximation. *J. Phys. Chem. Lett.* **2020**, *11*, 8208−8215
11. Garrity, K. F.; Bennett, J. W.; Rabe, K. M.; Vanderbilt, D. Pseudopotentials for high-throughput DFT calculations. *Comput. Mater. Sci.* **2014,** *81*, 446-452.
12. Huang, Y.; Nielsen, R. J.; Goddard, W. A., III Reaction mechanism for the hydrogen evolution reaction on the basal plane sulfur vacancy site of MoS_2 using grand canonical potential kinetics. *J. Am. Chem. Soc.* **2018,** *140*, 16773-16782.
13. Sumita, M.; Hu, C.; Tateyama, Y. Interface water on TiO_2 Anatase (101) and (001) surfaces: First-principles study with TiO_2 slabs dipped in bulk water. *J. Phys. Chem. C* **2010,** *114*, 18529-18537.
14. Tilocca, A.; Selloni, A. Structure and reactivity of water layers on defect-free and defective anatase TiO_2(101) surfaces. *J. Phys. Chem. B* **2004,** *108*, 4743-4751.
15. Rossmeisl, J.; Qu, Z. W.; Zhu, H.; Kroes, G. J.; Nørskov, J. K. Electrolysis of water on oxide surfaces. *J. Electroanal. Chem.* **2007,** *607*, 83-89.
16. Kim, J.; Chen, X.; Shih, P.-C.; Yang, H., Porous Perovskite-Type Lanthanum Cobaltite as Electrocatalysts toward Oxygen Evolution Reaction. *ACS Sustainable Chemistry & Engineering* **2017,** *5* (11), 10910-10917.
17. He, J. et al. Structure-property relationship of graphene coupled metal (Ni, Co, Fe) (oxy)hydroxides for efficient electrochemical evolution of oxygen. *J. Catal.* 2019, **377**, 619-628.
18. Bähr, A.; Moon, G.-h.; Tüysüz, H., Nitrogen-Doped Mesostructured Carbon-Supported Metallic Cobalt Nanoparticles for Oxygen Evolution Reaction. *ACS Applied Energy Materials* **2019,** *2* (9), 6672-6680.
19. Surendranath, Y.; Kanan, M. W.; Nocera, D. G., Mechanistic studies of the oxygen evolution reaction by a cobalt-phosphate catalyst at neutral pH. *J Am Chem Soc* **2010,** *132* (46), 16501-9.
20. Roy, C.; Sebok, B.; Scott, S. B.; Fiordaliso, E. M.; Sørensen, J. E.; Bodin, A.; Trimarco, D. B.; Damsgaard, C. D.; Vesborg, P. C. K.; Hansen, O.; Stephens, I. E. L.; Kibsgaard, J.; Chorkendorff, I., Impact of nanoparticle size and lattice oxygen on water oxidation on NiFeOxHy. *Nature Catalysis* **2018**, *1*, 820-829.
21. Li, P.; Jin, Z.; Qian, Y.; Fang, Z.; Xiao, D.; Yu, G., Probing Enhanced Site Activity of Co–Fe Bimetallic Subnanoclusters Derived from Dual Cross-Linked Hydrogels for Oxygen Electrocatalysis. ACS Energy Letters 2019, 4 (8), 1793-1802

22. Wu, L.; Li, Q.; Wu, C. H.; Zhu, H.; Mendoza-Garcia, A.; Shen, B.; Guo, J.; Sun, S., Stable Cobalt Nanoparticles and Their Monolayer Array as an Efficient Electrocatalyst for Oxygen Evolution Reaction. J Am Chem Soc 2015, 137 (22), 7071-4.
23. Li, Q.; Tang, S.; Tang, Z.; Zhang, Q.; Yang, W., Microwave-assisted synthesis of FeCoS2/XC-72 for oxygen evolution reaction. *Solid State Sciences* **2019,** *96*, 105968.
24. Su, H.; Zhao, X.; Cheng, W.; Zhang, H.; Li, Y.; Zhou, W.; Liu, M.; Liu, Q., Hetero-N-Coordinated Co Single Sites with High Turnover Frequency for Efficient Electrocatalytic Oxygen Evolution in an Acidic Medium. *ACS Energy Letters* **2019,** *4* (8), 1816-1822.
25. Ma, X.; Li, K.; Zhang, X.; Wei, B.; Yang, H.; Liu, L.; Zhang, M.; Zhang, X.; Chen, Y., The surface engineering of cobalt carbide spheres through N, B co-doping achieved by room-temperature in situ anchoring effects for active and durable multifunctional electrocatalysts. *J Mater Chem A* **2019,** *7* (24), 14904-14915.
